# Osmolyte Signatures for the Protection of *Aspergillus sydowii* Cells under Halophilic Conditions and Osmotic Shock

**DOI:** 10.3390/jof7060414

**Published:** 2021-05-26

**Authors:** Eya Caridad Rodríguez-Pupo, Yordanis Pérez-Llano, José Raunel Tinoco-Valencia, Norma Silvia Sánchez, Francisco Padilla-Garfias, Martha Calahorra, Nilda del C. Sánchez, Ayixón Sánchez-Reyes, María del Rocío Rodríguez-Hernández, Antonio Peña, Olivia Sánchez, Jesús Aguirre, Ramón Alberto Batista-García, Jorge Luis Folch-Mallol, María del Rayo Sánchez-Carbente

**Affiliations:** 1 Centro de Investigación en Biotecnología, Universidad Autónoma del Estado de Morelos (UAEM), Av. Universidad 1001, Col. Chamilpa, Cuernavaca C.P. 62209, Morelos, Mexico; eyarguez2013@gmail.com (E.C.R.-P.); yordanis.perezllano@yahoo.com (Y.P.-L.); rocio.rodriguez@uaem.mx (M.d.R.R.-H.); jordi@uaem.mx (J.L.F.-M.); 2 Centro de Investigación en Dinámica Celular, IICBA, UAEM, Av. Universidad 1001, Col. Chamilpa, Cuernavaca C.P. 62209, Morelos, Mexico; rabg@uaem.mx; 3 Instituto de Biotecnología, Universidad Nacional Autónoma de México (UNAM), Campus Morelos, Av. Universidad 1001, Col. Chamilpa, Cuernavaca C.P. 62210, Morelos, Mexico; raunel@ibt.unam.mx; 4 Instituto de Fisiología Celular, UNAM, Cto. Exterior s/n, Cd. Universitaria, Coyoacán, Ciudad de México C.P. 04510, Federal District, Mexico; nsanchez@ifc.unam.mx (N.S.S.); fpadilla@ifc.unam.mx (F.P.-G.); mcalahor@ifc.unam.mx (M.C.); apd@ifc.unam.mx (A.P.); asanchez@ifc.unam.mx (O.S.); jaguirre@ifc.unam.mx (J.A.); 5 Centro de Ciencias Genómicas, UNAM, Campus Morelos, Av. Universidad 1001, Col. Chamilpa, Cuernavaca C.P. 62210, Morelos, Mexico; nildita1985@gmail.com; 6 Catedras Conacyt-Instituto de Biotecnología, Universidad Nacional Autónoma de México (UNAM), Campus Morelos, Av. Universidad 1001, Col. Chamilpa, Cuernavaca C.P. 62210, Morelos, Mexico; ayixon.sanchez@ibt.unam.mx

**Keywords:** halophile, osmolyte, osmotic shock, HOG, *Aspergillus*, extremophile

## Abstract

*Aspergillus sydowii* is a moderate halophile fungus extensively studied for its biotechnological potential and halophile responses, which has also been reported as a coral reef pathogen. In a recent publication, the transcriptomic analysis of this fungus, when growing on wheat straw, showed that genes related to cell wall modification and cation transporters were upregulated under hypersaline conditions but not under 0.5 M NaCl, the optimal salinity for growth in this strain. This led us to study osmolyte accumulation as a mechanism to withstand moderate salinity. In this work, we show that *A. sydowii* accumulates trehalose, arabitol, mannitol, and glycerol with different temporal dynamics, which depend on whether the fungus is exposed to hypo- or hyperosmotic stress. The transcripts coding for enzymes responsible for polyalcohol synthesis were regulated in a stress-dependent manner. Interestingly, *A. sydowii* contains three homologs (Hog1, Hog2 and MpkC) of the Hog1 MAPK, the master regulator of hyperosmotic stress response in *S. cerevisiae* and other fungi. We show a differential regulation of these MAPKs under different salinity conditions, including sustained basal Hog1/Hog2 phosphorylation levels in the absence of NaCl or in the presence of 2.0 M NaCl, in contrast to what is observed in *S. cerevisiae*. These findings indicate that halophilic fungi such as *A. sydowii* utilize different osmoadaptation mechanisms to hypersaline conditions.

## 1. Introduction

Halophilic and halotolerant microorganisms, which thrive in saline environments, adapt strategies to cope with high concentrations of sodium chloride. The high ionic force in these environments provokes the inactivation of proteins and the osmotic pressure causes water loss from non-adapted cells, while the activation of stress-response pathways increases the levels of reactive oxygen species (ROS) [1,2]. Salt-adapted cells actively extrude metal cations to avoid toxicity and prevent water efflux by the accumulation of osmolytes, some of which also possesses ROS scavenging activity, among other mechanisms [1,2,3].

Osmolytes or compatible solutes are low molecular weight molecules that cells accumulate in high concentration to maintain osmotic balance [4,5,6,7]. Halophilic bacteria use glycine, betaine, and glutamate as the main osmolytes [8] whereas fungi preferably accumulate trehalose and poly-alcohols such as mannitol, glycerol, arabitol, and sorbitol [9,10,11].

The main pathway that regulates the production of osmolytes and other salinity responses in yeasts and filamentous fungi is the High Osmolarity Glycerol (HOG) signaling pathway [12,13,14]. This is a MAPK signal transduction cascade that leads to differential gene expression driven by Hog due to the phosphorylation of target transcription factors [12,13,15]. For example, the synthesis of osmolytes such as glycerol, arabitol, and mannitol is controlled by transcriptional regulation of the genes *gpd*, *ardh*, *mpdh*, and post-translational regulation of the activity of the proteins through phosphorylation [14,16,17,18].

Cellular responses in hyperosmotic media are well known in the halotolerant ascomycetous yeasts *S. cerevisiae* [19,20,21,22] and *Debaryomyces hansenii* [23,24,25,26] and in the halotolerant ascomycetous filamentous fungus *Aspergillus nidulans* [27]. In all these cases, the presence of salt entails stress responses that are superimposed with adaptation mechanisms to tolerate high salinity.

*Hortaea werneckii*, an extreme halotolerant black yeast, and *Wallemia ichthyophaga*, an obligate halophile, have recently emerged as models to study hyper-salinity adaptations in Basidiomycetes [10,23]. Osmolytes signatures, signaling pathways activation, cell wall adaptations, ionic balance, and membrane transporters have been studied in these models. However, previous studies in these and other halophilic fungi have been generally conducted in the minimum and maximum tolerated salinities [9,10,28]. Under these conditions, as in the studies of halotolerant fungi, the independent contribution of stress and salinity to physiological responses are difficult to discriminate.

We have recently shown that in the case of the halophilic ascomycetous model *A. sydowii*, the physiological responses to salinity vary if the fungus is under additional salinity stress [29]. In this strain, hyperosmotic conditions (2.0 M NaCl) induce the transcriptional regulation of cell wall reorganization, membrane cation transporters, hydrophobin production, and glycerol synthesis. However, such regulatory mechanisms were not observed at a salt concentration (0.5 M NaCl) that is optimal for the growth of this fungus [29]. Thus, we hypothesize that, at the optimal salinity, the fungus accumulates osmolytes other than glycerol, regulates ionic balance, and maintains low oxidative stress levels by mechanisms that do not require a steady transcriptional change. The aim of the present work was to analyze the osmolyte signatures in salt-adapted *A. sydowii* cells and their dynamic changes after hypoosmotic and hyperosmotic shocks.

In this report we examined osmolyte accumulation, transcriptional regulation of key enzymes involved in their synthesis, the activation of HOG signaling pathway, and the sodium and potassium ion balance in *A. sydowii* under hyperosmotic, hypoosmotic and optimal salinity conditions. Oxidative stress markers and antioxidant responses were also investigated to assert the level of stress in the evaluated conditions. This information will be useful for defining culture conditions in future biotechnological applications of *A. sydowii*. This fungus can grow with lignocellulosic substrates and hydrocarbons as the only carbon sources [29,30,31,32], and produces biotechnologically relevant enzymes [33,34,35,36,37] and secondary metabolites [38,39,40], which attest to its potential use as a tool in biorefineries and in bioremediation approaches.

## 2. Materials and Methods

### 2.1. Fungal Strain and Culture Conditions

Isolation of the moderate halophilic fungus *A. sydowii* strain BMH0004 has been previously described [29,30]. The strain was maintained in PDA petri dishes grown at 28 °C and stored at 4 °C or as spores suspension in 20% glycerol supplemented with 0.5% NaCl at −80 °C in the Fungal Culture Collection of the Center for Research on Biotechnology (CEIB, UAEM, Morelos, Mexico) with reference number BMH0004, in the Technological University Collection of Industrially Relevant Microorganisms (TUCIM, Vienna, Austria) with reference number 6524 and the Ex Culture Collection of the Infrastructural Centre Mycosmo (MRIC, UL, Ljubljana, Slovenia) with reference number EXF-12860.

For all experimental determinations, *A. sydowii* was grown in liquid mineral medium with glucose (MMG medium) and incubated at 28 °C and 150 rpm. The MMG medium (containing: 7.8 mg/L CuSO_4_·5H_2_O, 18 mg/L FeSO_4_·7H_2_O, 500 mg/L MgSO_4_·7H_2_O, 10 mg/L ZnSO_4_, 50 mg/L KCl, 1 g/L K_2_HPO_4_ and 2 g/L NH_4_NO_3_) was adjusted to pH 6 with H_3_PO_4_ or KOH, then sterilized by autoclaving and supplemented with 0.5 M or 2.0 M NaCl and 2% filter-sterilized glucose.

### 2.2. Growth Rate Determination

To determine the growth rate of *A. sydowii* at different salinities, 10^6^ spores were inoculated into 250 mL flasks with 50 mL of MMG with different concentrations of NaCl (without NaCl, with 0.5 M, 1.0 M, or 2.0 M NaCl). The cultures were incubated at 28 °C with constant shaking at 150 rpm. All the mycelium in a culture flask was harvested every 2 days until no changes in biomass were recorded. The mycelium was collected by filtration with a 40 µm pore size cell strainer, dried at 60 °C in an oven and weighted. All experimental determinations were made in triplicate.

### 2.3. Identification and Quantification of Compatible Solutes

Intracellular glycerol, erythritol, ribitol, xylitol, arabitol, galactitol, sorbitol, mannitol, maltitol, and trehalose (Standards kit, Cat. 47266, Sigma-Aldrich, St. Louis, MO, USA) were measured by HPLC (Supplementary Figures S1 and S2). The extraction of the metabolites was carried out by a modification of the Bligh and Dyer method [9,41]. Briefly, 100 mg of mycelium were suspended in 3680 μL of Bligh and Dyer solution (chloroform–methanol–water (10:5:3.4)) and stirred vigorously for approximately 30 min. 433 µL of chloroform and 433 μL of demineralized water were added, and the suspension was incubated for 30 min with stirring. The samples were centrifuged at 5500× *g* for 10 min for phase separation. The upper methanol-water phase was collected and stored at −20 °C until analysis.

The HPLC analysis was performed in an isocratic system with an AMINEX-HPX87H column (300 mm × 7.8 mm, Bio-Rad, Munich, Germany) at 50 °C. Injection volume was 50 μL for all samples. Calibration curves of standards were obtained using 50, 200, 400, 600, and 800 µg/mL of each compound. The separation was carried out by elution with 5 mM sulfuric acid, at a flow rate of 0.8 mL/min. Chromatogram analysis was performed using ChromQuest software v2.51 (Thermo Fisher Scientific, Waltham, MA, USA).

### 2.4. Compatible Solutes in Salt-Adapted Mycelium

To determine the compatible solutes in cells adapted to salt, 10^6^ spores were inoculated into 250 mL flasks with 100 mL of MMG with different concentrations of NaCl (0 M, 0.5 M, or 2.0 M NaCl). The mycelium was collected at different time points of the growth curve (5, 7, and 11 days) and dried until constant weight, in an oven at 60 °C, before extraction of compatible solutes as previously described. All the experiments were performed with three culture replicates.

### 2.5. Compatible Solutes after Osmotic Shock

Compatible solutes were determined in cells after osmotic shock exerted by transferring the mycelium in all possible conditions between 0 M, 0.5 M, and 2.0 M NaCl. The fungus was cultured in 500 mL flasks with 200 mL of MMG with or without NaCl and incubated for 7 days at 28 °C, 150 rpm. The obtained pre-inoculum was harvested using a 40 μm pore size cell strainer and 1 g of wet biomass was transferred to 250 mL flasks with 50 mL of MMG with or without NaCl and incubated at 28 °C, 150 rpm. The mycelia were collected after 10 min, 30 min, 2 h, 8 h, 24 h, and 48 h post-inoculation, dried in an oven at 60 °C and compatible solutes were extracted as described above. All the experiments were performed with three culture replicates.

### 2.6. RNA Extraction and qPCR Analysis

The fungus was cultured for 7 days as previously described (t0) and was subjected to osmotic shock following the same procedure as for the determination of compatible solutes. The mycelium was harvested by centrifugation, frozen with liquid nitrogen, grinded using a mortar and pestle, and 100 mg were used to isolate total RNA using the TRI-reagent method (Sigma-Aldrich). cDNA was synthesized from 2 µg of DNase-treated total RNA, using the RevertAid™ H Minus First Strand cDNA synthesis kit with a dT18 primer (Thermo Fisher Scientific).

Primers for qPCR analysis were designed using Primer3Plus [42] and their physico-chemical properties and amplicon structures were evaluated using DINAMelt [43] and Mfold [44], respectively. All primers used are listed in Supplementary Table S1.

Two-step qPCR reaction conditions (temperature, primer concentration and efficiency) were optimized for each primer pair (see Supplementary Table S1 for reaction conditions). qPCR reactions contained 5 µL of QuantiNOVA SYBR GREEN Master Mix (QIAgen, Hilden, Germany) and 1 µL of a 1:8 dilution of cDNA in a final volume of 10 µL. All reactions were quantified in duplicate using a Rotorgene apparatus (QIAgen, Hilden, Germany). A melting curve and a polyacrylamide gel electrophoresis were used to verify the specificity of the amplified product.

Relative expression levels were calculated with the Pfaffl method using the REST software [45,46]. In cases where the randomization test performed by REST confirmed statistical significance of the fold change, a binary logarithm (logFC) over 2 was considered as biologically significant up- or down-regulation. The genes *sarA* and *cox5* were used as reference genes for normalization [47].

### 2.7. Phylogenetic Analysis of MAPK Orthologs

Protein sequences corresponding to mitogen-activated protein kinase (MAPK) orthologs (KOG0660) were retrieved from the genomes of Aspergilli in the Mycocosm database by KOG annotation search. These sequences were aligned using the MUSCLE algorithm with default parameters [48] as implemented in the MEGA suite [49]. The resulting multiple sequence alignment (MSA) was edited in AliView [50] to remove non parsimonious-informative sequence blocks, resulting in a MSA with 253 sites. For phylogenetic reconstruction, the best substitution model was selected in MEGA based on the lowest BIC scores (Bayesian Information Criterion). Phylogenetic distances were inferred by using the Maximum Likelihood (ML) method and Le_Gascuel_2008 model [51] assuming gamma distributed evolutionary rates among sites. The ML Tree confidence was evaluated with the Bootstrap method using 1000 iterations [52]. The bootstrap consensus tree was modified for visualization using FigTree (http://tree.bio.ed.ac.uk/software/figtree/, accessed on 15 May 2021).

### 2.8. Western Blot of Phosphorylated Hog

Cultures of *A. sydowii* were subjected to osmotic shock as previously described. The mycelium was harvested with a cell strainer after 5, 15, 30, 60, 120, and 180 min post-inoculation, and treated with 85% trichloroacetic acid for 10 min [53]. The fixed cells were washed three times with distilled water, frozen and macerated with liquid nitrogen, and vortexed with lysis buffer (500 mM Tris pH 6.8, 100 mM DTT, 2% SDS, 4% glycerol and 0.01% bromophenol blue) and 0.5 mm diameter glass beads [53,54]. A constant volume of protein extract was used for Western blot. PVDF membranes were blocked with 7% skim milk (BD Bioscience, Franklin Lakes, NJ, USA) in Phosphate Buffer Saline (PBS), followed by incubation with an anti-p38 phosphorylated MAP kinase antibody (Cat. 4511, Cell Signaling, Danvers, MA, USA) at a 1:1000 dilution. Anti-Hog antibody (Cat. SC-9079, Santa Cruz, Dallas, TX, USA) was used at a 1:1000 dilution to detect total protein and served as loading control. An anti-rabbit IgG coupled to HRP (Invitrogen, Waltham, MA, USA) was used at a 1:10,000 dilution and incubated at room temperature for 1 h. The molecular weight marker used in this assay was Page Ruler pre-stained TM (10–180 kDa, ThermoScientific, Waltham, MA, USA). A protein extract from *S. cerevisiae* strain BY7472 was used as a positive control. In this case, *S. cerevisiae* cells were grown overnight in YPD broth, subjected to 1.0 M NaCl osmotic shock for 10 min, and treated as previously described for fungal mycelium [53,54].

### 2.9. Na^+^/K^+^ Quantification

For the quantification of intracellular sodium and potassium, 300 mg of wet mycelium (5, 7, and 11 days of culture, as previously described) was washed two times with deionized water and vacuum dried using Whatman filters with a pore size of 0.45 µm in a Millipore Multifilter equipment. The biomass was resuspended in 5 mL of deionized water and homogenized with a Teflon pestle in a tissue homogenizer for approximately 2 min. The homogenate was collected by rinsing the tissue homogenizer with 5 mL of deionized water to obtain a final volume of 10 mL. From this volume, 5 mL were heated in a water bath at the boiling point for 20 min and then centrifuged for 5 min at 1625× *g*. The supernatant was collected and Na^+^ and K^+^ were quantified on a Flame Photometer (Carls Zeiss PF5 371777) [55]. For the quantification, 1 mM NaCl and 1 mM KCl were used as standards, corresponding to 100 AU. All measurements were made in triplicate.

### 2.10. Quantification of Oxidative Stress Markers and Antioxidant Responses in A. sydowii

The mycelium was collected in a cell strainer and macerated with liquid nitrogen using a mortar and pestle. Cell lysis was achieved by adding 200 µL of lysis buffer (0.3 M Tris pH 6.8, SDS 2% and glycerol 4%) to 100 mg of frozen macerated mycelium, with 0.3 g of 0.5 mm diameter glass beads. Samples were vortexed four times on lapses of 1 min and allowed to rest for another minute on ice. The supernatant was recovered by centrifugation and stored at −80 °C until analysis.

Hydrogen peroxide was quantified as total peroxides in cell extracts using the BIOXYTECH^®^ H2O2-560™ Assay kit (Cat. 21024, OXIS International Inc., Portland, OR, USA). Briefly, this colorimetric assay is based on the oxidation of ferrous ions, which then bind to the xylenol orange dye to yield a colored complex. Sorbitol in the reaction enhances the oxidation of ferrous ions, which increases the assay sensitivity. The indicator dye production was evaluated by absorbance at 560 nm.

Protein Advanced Oxidation Product (PAOP) level was determined by a modified Witko’s method [56] using chloramine-T (*N*-chloro-p-toluene-sulfonamide) as standard. Chloramines were determined by production of triiodide ion from the oxidation of potassium iodide in solution at 340 nm.

Lipid peroxidation was evaluated by the quantification of malondialdehyde (MDA) and 4-hydroxyalkenals (4-HDA), two common degradation products of lipid peroxidation. MDA and 4-HDA were determined by reaction with *N*-methyl-2-phenylindole to form a chromophoric cyanine that can be quantified spectrophotometrically at 586 nm [57]. MDA concentration was calculated using a standard curve. To further determine cell susceptibility to lipid peroxidation, cell extracts were incubated with 2 mM copper sulphate at 37 °C for 24 h [58]. At the end of the incubation period, MDA and diene conjugate levels were also measured.

Superoxide dismutase (SOD) activity was determined according to the Marklund method [59] based on the ability of the enzyme to inhibit the autoxidation of pyrogallol. The rate of autoxidation is obtained from the increase in absorbance at 420 nm, in the absence of superoxide dismutase. The levels of reduced glutathione (GSH) present in the sample were determined as described previously by [60]. Briefly, the GSH reacts with 5,5′-Dithiobis-(2-nitrobenzoic acid) dye (DTNB dye) to yield a colored compound that absorbs light at 412 nm. Concentration of GSH was determined by comparing the samples with a standard curve.

### 2.11. Statistical Analysis

Results are expressed as means ± SD. Statistical analysis of solute concentration was performed using one-way ANOVA considering a fixed-effect model with salinity as predictor variable. The ANOVA premises were assessed according to the Kolmogorov-Smirnov normality test [61] and the Levene or Brown-Forsythe variance homogeneity test [62]. The means of multiple comparison tests were performed by Duncan test [63]. Where the normality and homoscedasticity requirements were not verified, a Kruskal-Wallis test was performed, and the multiple mean comparisons were performed using the Dunn’s test. The level of significance was set at α = 0.05 using the STATISTICA software, v. 7.0, (StatSoft, Inc., Tulsa, OK, USA).

## 3. Results and Discussion

### 3.1. Compatible Solutes in Salt-Adapted A. sydowii Cells

**Figure 1:**
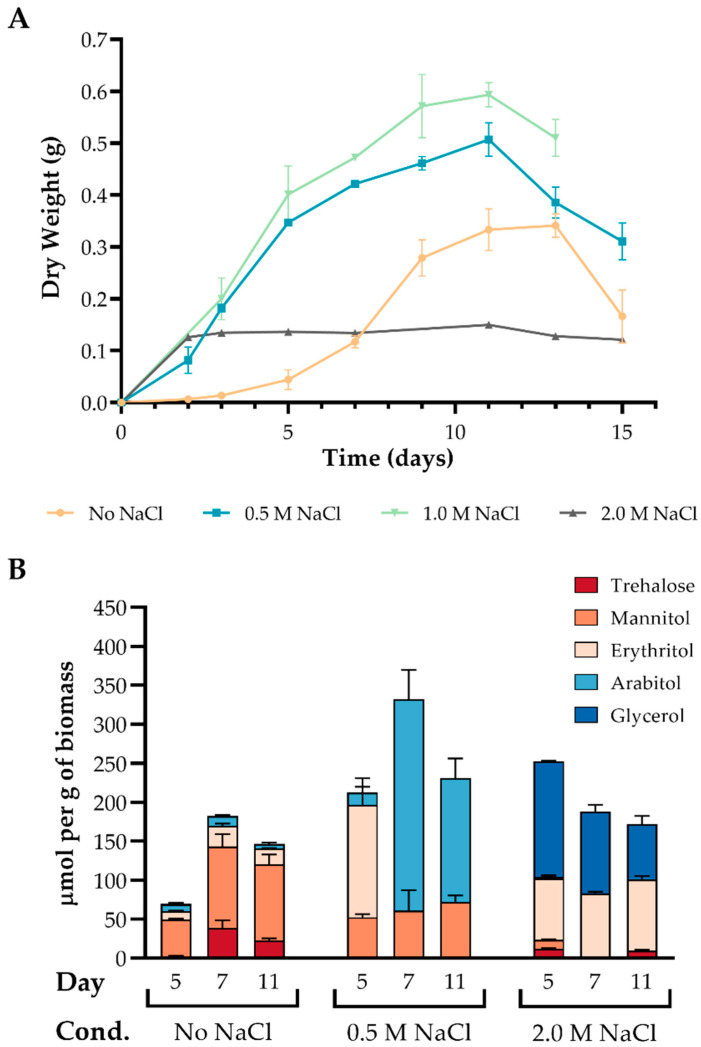
Growth rate of *A. sydowii* (**A**) and osmolyte accumulation (**B**) in optimal, hypo- and hyper osmotic conditions. Data are means ± SD calculated from three independent experiments (*n* = 3). Statistical analyses are detailed in Supplementary Table S3.

To avoid the stress induced by nutritional deprivation, we cultured the fungus with glucose as carbon source [30,32]. In a shaken flask cultivation, most microorganisms will grow exponentially until the stationary phase, where active growth ceases due to nutrient scarcity and other biologically imposed limits. The metabolic response of the cells is expected to be different in each growth phase, and therefore the growth rate of *A. sydowii* in different salinities was determined to establish the time needed to analyze the osmolyte content (Figure 1A). The duration of the growth phases was different between the tested conditions, reinforcing the notion that there are three different biological scenarios: the fungus growing under optimal salinity condition, or under either hyperosmotic or hypoosmotic conditions. An initial adaptation (or lag) phase was evident in the condition without NaCl, while this phase was nearly inexistent in the other three conditions. The exponential growth phase lasted until day 9 of culture under the optimal conditions and without NaCl, but only lasted until day 2 at 2.0 M NaCl. The doubling time of *A. sydowii* on 0.5 M and 1.0 M NaCl were 10 and 11 h, respectively, while in the absence of salt and 2.0 M NaCl were 13.5 and 14.6 h, respectively. In further experiments we used 0.5 M NaCl as the optimal salinity condition.

These results confirm that *A. sydowii* is a moderate halophile with optimal growth in the salinity range 0.5 to 1.0 M NaCl (Figure 1A and Supplementary Table S2). Previous reports of marine *A. sydowii* strains isolated from corals reinforce the notion that this fungus thrives in salinities near the seawater NaCl concentration (0.6 M NaCl) [64,65]. In this environment the fungus infects corals causing tissue purpling and galling. Although *A. sydowii* has a terrestrial origin, marine isolates have caused a pandemic that has reduced the coral reef population [64,65]. Nevertheless, terrestrial isolates, as is the case of the strain BMH-0004 analyzed in this study, are not pathogenic to corals [64,65]. 

Osmolyte accumulation in *A. sydowii* was evaluated at three time points: 5-, 7-, and 11-days post-inoculation, which cover different states of growth under all conditions. Given the unique profiles of the growth curves, it was difficult to determine equivalent physiological states for the cultures. At days 5 and 7, the fungus is in exponential growth phase in the cultures without NaCl or with 0.5 M NaCl. At day 11, all the cultures had reached the stationary phase. In the cultures with 2.0 M NaCl, the exponential growth phase was not sampled because the mycelium grows in a small window of time until a stationary state where the fungus remains viable but ceases to grow (Figure 1A). This pattern is similar to the growth of fungi such as *Rhizopus microsporus* and *A. fumigatus* on nutritionally poor media [66].

It has been proposed that under osmotic stress, cells respond with an increased synthesis of compatible solutes, regardless of the solute, causing the lowering of water activity [67]. In *A. sydowii* the largest accumulation of compatible solutes (mannitol, erythritol and arabitol) was observed under optimal conditions of growth (Figure 1B). This result contradicts the general notion that osmolytes are mainly accumulated under hyperosmotic conditions and cellular stress [11,67,68,69,70,71]. However, it has been documented that other fungi exhibit higher polyol concentration when growing on their optimal NaCl and KCl concentration [72].

Our results also showed that *A. sydowii* increased the concentration of erythritol and glycerol only when the cells were exposed to hyper-salinity (Figure 1B). In the media without salt, mannitol was the most abundant metabolite followed by trehalose and erythritol. In the optimal condition there is an apparent shift in the physiological accumulation of osmolytes after 5 days of culture, as mannitol and erythritol were present at day 5, while arabitol replaces erythritol at days 7 and 11. Interestingly, glycerol was detected only in the hypersaline condition, followed by erythritol and trace amounts of trehalose and mannitol (Figure 1B). Under a hyperosmotic stimulus, *W. ichthyophaga* also synthetizes glycerol and arabitol [11], while *Trichosporonoides megachiliensis* and *H. werneckii* synthetize glycerol and erythritol [73], and *A. tamari*, *A. montevidensis*, and *A. wentii* synthetize mainly glycerol [72,74,75].

### 3.2. Transcriptional Profiling of Genes Coding for Osmolyte Synthesis Enzymes

**Figure 2:**
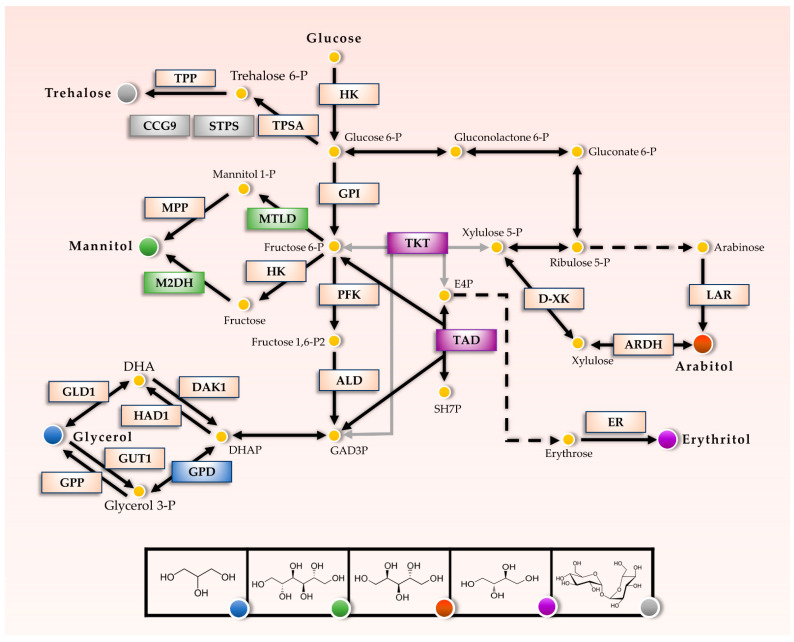
Pathways for the synthesis of metabolites with possible osmolyte functions in response to salinity. The pathway has been modified from the *Aspergillus nidulans* KEGG pathway. Enzymes involved in the regulation of osmolyte concentration and depicted with the color grey: trehalose pathway; green: mannitol; blue: glycerol; purple: erythritol, and red: arabitol were evaluated by qPCR. The enzymes depicted in light orange were not evaluated. TPSA (Trehalose phosphate synthase), STPS (Heat shock trehalose phosphate synthase), CCG-9 (Trehalose phosphate synthase), TPP (Trehalose-6-P phosphatase), MTLD (Mannitol-1-phosphate 5-dehydrogenase), M2DH (Mannitol 2-dehydrogenase), MPP (Manitol/Hexitol phosphatase), HK (Hexokinase), GPI (Phospho-glucose isomerase), PFK (Phospho-fructokinase), ALD (Aldolase), GPD (Glycerol-3-P dehydrogenase), GPP (Glycerol-3-P phosphatase), GUT1 (Glycerol-kinase), HAD1(Halo-acid dehalogenase), DAK1 (Dihydroxyacetone kinase) GLD1 (Glycerol dehydrogenase). TKT (Transketolase), TAD (Trans-aldolase), LAR (L-arabinose), ARDH (L-arabinitol dehydrogenase), D-XK (D-Xylose Kinase), ER (Erythrose reductase).

**Figure 3:**
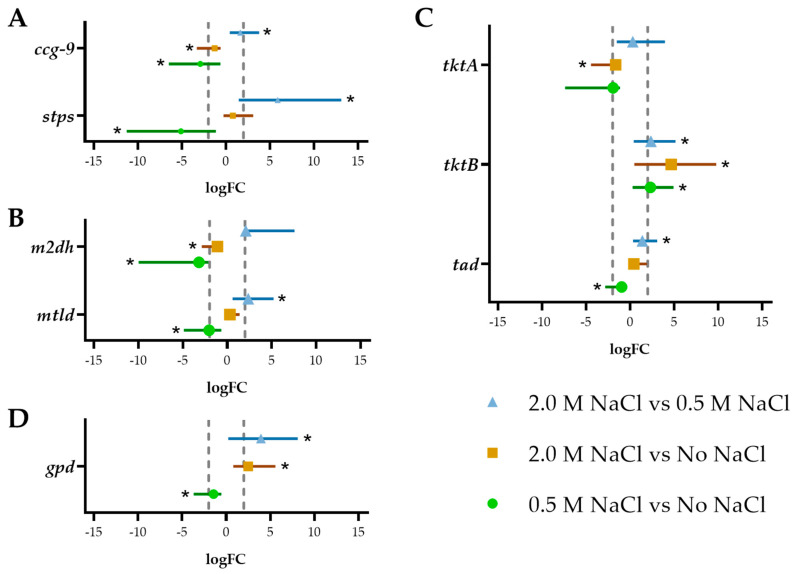
Expression analysis of transcripts related to osmolytes synthesis of *A. sydowii* in different salinities after 7 d of culture. The values correspond to the average and standard deviations of three biological replicates (*n* = 3) and two technical qPCR replicates. Analyzed genes were grouped according to the pathway: (**A**) Trehalose synthesis, (**B**) Mannitol synthesis, (**C**) Pentose phosphate pathway (for arabitol and erythritol synthesis) and (**D**) Glycerol synthesis. Statistical significance (*) was assessed by a randomization test performed with the software Rest [46]. The dashed vertical lines correspond to a cutoff logFC = 2.

The metabolic pathways of osmolyte synthesis have been described in several fungi including model Aspergilli such as *A. niger*, *A. nidulans*, and *A. fumigatus* [76,77,78,79,80]. However, the genetic regulation of these pathways is diverse, and hence, a characterization of the transcriptional regulation of genes coding for osmolyte synthesis enzymes should be conducted in distinct species and under different conditions. The metabolic pathways leading to the synthesis of the identified osmolytes are depicted in Figure 2, highlighting the enzymes for which transcriptional regulation was evaluated (Figure 3).

Trehalose, a glucose disaccharide, is synthesized from glucose-6-phosphate by two consecutive reactions catalyzed by the trehalose-6-phosphate synthase (TPS) and trehalose-6-P phosphatase (TPP) enzyme complex. In *S. cerevisiae* the genes *tps1* and *tps2* encode the TPS and TPP subunits, respectively, while the genes *tps3* and *tsl1* encode regulatory subunits. In *A. nidulans* the orthologues of the *tps1-3* genes are named *tpsA*, *orlA*, and *tps3*. All three genes contain a glycosyl transferase domain from family 20 (GT 20 domain, pfam: PF00982), while *orlA* and *tps3* additionally contain a TPP domain (pfam: PF02358). *A. nidulans* has also a heat shock trehalose synthase gene (*stps*) that has been found in Aspergilli, a homolog of the *Neurospora crassa ccg-9* gene which encodes a trehalose synthase with a glycosyl transferase family 1 domain, and a trehalose-6-P phosphatase gene containing only the TPP domain, which is putatively involved in the synthesis of trehalose from trehalose-6-P. The *A. sydowii* homologs of these genes are listed in Supplementary Table S4.

Among the tested metabolites in *A. sydowii*, we found that trehalose was accumulated to a lesser extent (Figure 1B). The highest intracellular concentration of trehalose occurred without NaCl at day 7 (38 µmol/g) ant it was also produced in the condition with 2.0 M NaCl (at days 5 and 11). As shown in Figure 3, this is consistent with the expression of the *stps* trehalose synthase gene, which was upregulated in both extreme conditions, but downregulated in optimal salinity. The *ccg*-9 gene has the same expression pattern but with lesser differential expression among the conditions (Figure 3A).

Trehalose is suggested to function as a reserve carbohydrate, as an osmo-protectant, or for protection against protein denaturation by dehydration [81,82]. For example, spores that have a high content of trehalose are more resistant to temperature stress, dehydration, freezing, oxidizing agents, or starvation [81,83,84,85,86]. *A. nidulans* and *S. cerevisiae* mutants lacking the *tps1* gene are also less thermo- and halotolerant [69,86,87]. Nevertheless, *tps* genes are also involved in the regulation of cell wall structure by modulating chitin synthase activity [88,89,90,91], hence the effect of these genes might intertwine both physiological responses. In *A. sydowii*, the low amount of accumulated trehalose and the high differential expression of the *stps* and *ccg*-9 genes suggest that they are involved in stress responses other than trehalose synthesis.

The most common pathway of mannitol biosynthesis in filamentous fungi is the conversion from fructose-6-P to mannitol-1-P by the mannitol-1-P dehydrogenase and subsequently the dephosphorylation to obtain mannitol [92]. An alternative pathway converts fructose to mannitol by the mannitol dehydrogenase. In *A. nidulans* the genes encoding these enzymes are named *mtld* and *m2dh*, respectively. The patterns of expression of *mtld* gene and trehalose synthase genes were similar, showing higher expression in extreme conditions when compared to optimal salinity (Figure 3B). However, *mtld* expression did not correlate with mannitol accumulation particularly without NaCl or 0.5 M NaCl. The *m2dh* gene, on the other hand, is highly expressed in the condition without NaCl, but it is downregulated in 0.5 M NaCl (Figure 3B). The expression pattern of these genes does not account for the accumulation of mannitol in 0.5 M NaCl.

Although mannitol has been found in high abundance in several fungal species, accumulating evidence suggests that it is not required in all fungi for osmotic stress protection, oxidative stress prevention, or sporulation [92]. Therefore, while its role in fungal physiology is not completely clear, mannitol is not an essential polyol in fungi [93]. In *A. sydowii,* mannitol accumulation might result from active growth metabolism, as it does not seem to be regulated by salt stress response mechanisms.

As mentioned before, glycerol is the canonical osmolyte produced under hyperosmotic stress by many microorganisms. Glycerol is produced by two routes from the dihydroxyacetone phosphate (DHAP) obtained during glycolysis. The first pathway involves the conversion of DHAP to glycerol-3-P by the glycerol-3-P dehydrogenase (GPD) enzyme, which is the main regulatory point in the glycerol pathway. Glycerol-3-P is then converted to glycerol by the glycerol-3-P phosphatase (GPP), while the opposite reaction is catalyzed by the glycerol-kinase (GUT1). The alternative pathway involves the conversion of DHAP to dihydroxyacetone (DHA) by a halo-acid dehalogenase (HAD1) and the subsequent conversion to glycerol by the glycerol dehydrogenase (GLD1).

In *A. sydowii* the *gpd* gene was highly expressed under hyperosmotic conditions, but not under the optimal salinity (Figure 3D). This coincides with the glycerol accumulation in 2.0 M NaCl. In fact, the *gpd* gene was downregulated in the optimal condition when compared to the condition without salt. This reinforces our hypothesis that osmotic stress responses are not taking place when *A. sydowii* is grown under optimal salinity but occur only under the hyperosmotic stress induced by 2.0 M NaCl.

While trehalose, mannitol, and glycerol are directly under the influence of the regulatory frame of glycolysis, erythritol and arabitol are produced from pentose phosphate pathway intermediates and are therefore produced preferentially in conditions where the energy and redox balance in the cell favors anabolic reactions. Arabitol is produced from L-arabinose by the L-arabinose reductase (LAR) or from L-xylulose by a reversible reaction catalyzed by the L-arabitol dehydrogenase (ARDH). A key enzyme controlling the flux to xylulose is the transketolase which is encoded by the gene *tkt*. In *A. sydowii* we found two homologs of this gene, named *tktA* and *tktB*. The former was downregulated in 2.0 M NaCl, while the latter had an expression that increases with increasing salinity (Figure 3C). Neither of these expression patterns correlated with erythritol or arabitol accumulation.

On the other hand, erythritol is produced from erythrose by the erythrose reductase (ER), which in *A. niger* was identified as an aldehyde reductase with a broader specificity for other five-carbon aldehydes [94]. The first step in the shunt from glycolytic intermediates to erytrose-4-P is carried out by the enzyme trans-aldolase (TAD) and it is, therefore, a possible point of regulation of this pathway. The *tad* gene in *A. sydowii* did not show a high differential expression in the salinity conditions tested, and therefore was not analyzed further.

Compared to glycerol, erythritol has lower hygroscopicity and antioxidant properties [73]. Both low-molecular-weight polyols are more effective in osmotic protection than mannitol or arabitol, which have higher molecular size. Some studies have reported that osmolytes with higher-molecular-weight can even inhibit enzymatic activity as compared with the same concentration of glycerol [67,95,96]. Besides, there is an added “carbon cost” in the synthesis of larger osmolytes, which the cell may not afford if it is under stress.

Consistent with our results, *Hortaea werneckii*, *Penicillium chrysogenum*, and some Aspergilli also accumulate mannitol, arabitol, and erythritol at the optimal growth condition. However, a contrasting observation with our results was obtained for *A. oryzae*, *A. fischeri*, and *A. niger*, where trehalose was the most abundant osmolyte [3,97]. As indicated, mannitol was the most abundant polyol in *A. sydowii* BMH004, found in different days of growth, both in absence of salt and under optimal salinity (Figure 1B). Consistently, in *P. chysogenum* and *A. niger* in the absence of salt, mannitol is accumulated in greater proportion, and arabitol is only detected in minimal quantities [97]. It is known that this polyol can protect against the inactivation of enzymes by heat [98], but due to its limited solubility and tendency to crystallize, mannitol provides little protection against osmotic stress and freezing [99,100,101].

In contrast, at higher salinity *H. werneckii* (4.28 M NaCl) and *W. ichthyophaga* (4.25 M NaCl) accumulate mainly glycerol and lower amounts of erythritol [9,11]. Our results show that *A. sydowii* accumulated both polyols in similar concentrations (82.9 and 105.27 µmol/g at day 7) when the fungus is growing at 2.0 M of NaCl (Figure 1). In several reports of different yeasts and fungi, glycerol was the main solute accumulated in response to hyperosmotic stress [102,103]. For instance, *Yarrowia lypolitica* also accumulates erythritol in higher concentrations than mannitol when exposed to high osmotic pressure [104].

### 3.3. Dynamics of Compatible Solutes Accumulation after Hypoosmotic or Hyperosmotic Shock

In model fungi, the accumulation of compatible solutes has been analyzed during hyperosmotic or hypoosmotic shock but there are few studies analyzing halophile fungi growing under optimal salinity conditions and changed to osmo-stressful conditions [105,106]. Here we have termed these as stress-inducing shocks to highlight the difference from hyperosmotic and hypoosmotic shocks between non-optimal growth conditions. We evaluated the accumulation of polyols and trehalose when *A. sydowii* was growing with 0.5 M NaCl and then exposed to a medium without salt or with 2.0 M NaCl.

After a hyperosmotic shock, *S. cerevisiae*, *H. werneckii*, and *W. ichthyophaga* prevalently accumulate glycerol. However, in *A. sydowii* cells under stable salinity conditions, we observed that the accumulation of glycerol occurred only at 2.0 M NaCl. This leads to questions as to whether glycerol or other osmolytes will be produced if mycelium is shifted from hypoosmotic conditions (without NaCl) to optimal salt conditions (0.5 M NaCl), which presumably do not represent a stressful condition for this fungus. Therefore, we determined the accumulation of compatible solutes of *A. sydowii* when exposed to hypoosmotic, hyperosmotic, and stress-inducing osmotic shocks.

**Figure 4:**
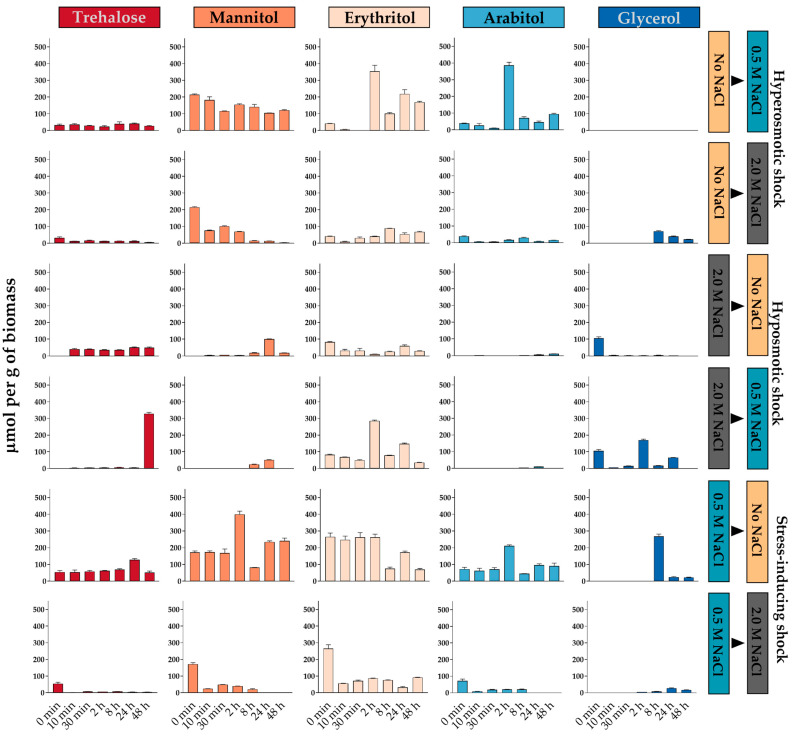
Accumulation of compatible solutes in *A. sydowii* after different osmotic shocks. The labels on the right indicate the culture conditions before and after the osmotic shock. The data represent the average and standard deviation of at least three replicates (*n* = 3). Statistical analyses of the data are summarized in Supplementary Table S5.

**Figure 5:**
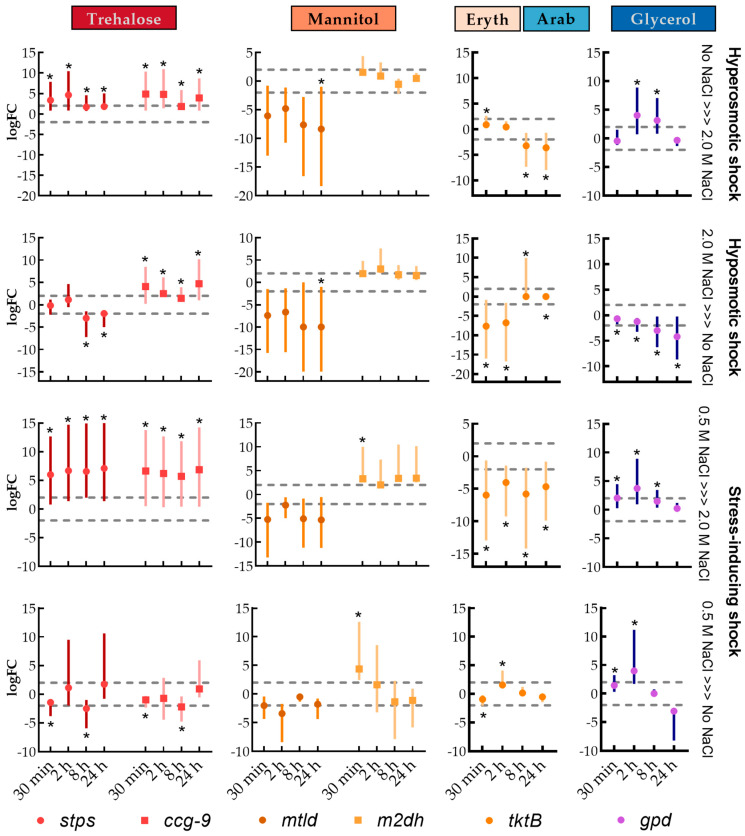
Transcriptional regulation of enzyme genes involved in the synthesis of osmolytes after hyperosmotic shock, hypoosmotic shock, and stress-inducing shock. The values correspond to the average and standard deviations of three biological replicates (*n* = 3) and two technical qPCR replicates. The statistical significance (*) was assessed by a randomization test performed with the software Rest [46]. The dashed horizontal lines correspond to a cutoff logFC = ±2.

When cultures without NaCl were transferred to 0.5 M NaCl (Figure 4), the fungus responded after 2 h with a spike in the concentration of erythritol and arabitol, which diminished after 8 h to the levels encountered in mycelia cultured at 0.5 M NaCl. Trehalose and mannitol levels did not change, and glycerol was not produced, even though similar salinities induce its accumulation in other non-halophile Aspergilli such as *A. nidulans* and *A. niger* [4,107]. In contrast, the shifting of cultures without NaCl to 2.0 M NaCl induced a small glycerol accumulation after 8 h and a progressive decrease of mannitol concentration. This osmotic shock induced the expression of both *stps* and *ccg-9* genes (Figure 5), which did not correlate with trehalose accumulation. The gene *m2dh* did not change its expression, while the *mtld* gene was downregulated after the shock, coinciding with the decrease of mannitol concentration. The *gpd* transcripts were detected transiently between 2 and 8 h, which also coincides with the increase in glycerol after 8 h. These observations are different than in studies performed in *S. cerevisiae* and *D. hansenii*, and others, where the intracellular concentration of glycerol increased after 30 min when the cells were exposed to a moderate osmotic shock (without salt to 0.5 M of NaCl) [54,108,109,110].

After a hypoosmotic shock from 2.0 M NaCl to a medium without salt, trehalose was detected as early as 10 min, while glycerol rapidly disappeared, but there were not dramatic changes in total osmolyte concentration (Figure 4). Under this shock, the *ccg-9* gene was upregulated soon after, while the *stps* gene was downregulated after 8 h and the *gpd* transcript levels decreased progressively (Figure 5). These changes alone do not explain the dynamics of trehalose and glycerol but are consistent with the physiological response of the mycelium in this scenario. When the hypoosmotic shock was from 2 M NaCl to a medium with 0.5 M NaCl, fluctuations were more evident, as erythritol concentrations spiked after 2 h and glycerol initially disappeared and later spiked also after 2 h from the shock. There was a spike of trehalose 48 h after this treatment for which we have no plausible explanation.

Our results indicated that, when changed to a condition without salt, the concentrations of mannitol and arabitol spiked transiently at 2 h and returned to approximately the initial level after 24 h (Figure 4), which coincided with an initial upregulation of *m2dh* and to a lesser extent *tktB* (Figure 5), but these might not be responsible for the observed osmolyte dynamics. The concentration of glycerol spiked at 8 h (263 µmol/g dry mass) and was still detected 48 h after this hypoosmotic shock, coinciding with the transient upregulation of *gpd* (Figure 5). The activation of *gpd* gene and the subsequent production of glycerol is not generally regarded as a response to hypoosmotic shock, but in this case, it highlights the notion that this mechanism is associated with osmotic stress regardless of the direction of the stimulus.

When changed from the optimal salinity to the hypersaline condition, instead of the expected increase in the concentration of compatible solutes, there was a reduction in trehalose, mannitol, erythritol, and arabitol and only a slight increase in glycerol (Figure 4). The *mtld* and *m2dh* genes were highly upregulated shortly after the shock, and remained so even after 24 h, which does not explain mannitol dynamics. The *tktB* gene was significantly downregulated shortly after the hypoosmotic shock, which might account for the reduction of erythritol and arabitol. The *gpd* gene was transiently upregulated between 30 min and 8 h after the shock, preceding the small surge of glycerol in this condition (Figure 5). The physiological responses of *A. sydowii* to hyperosmotic changes in the medium are similar in terms of accumulated osmolytes and enzyme gene regulation but are counterintuitively more pronounced in the change from 0.5 M NaCl than from the medium without salt.

Altogether, these observations show that the response of this fungus is different from other halotolerant and halophile model fungi. Moreover, we observed that the accumulation of glycerol in *A. sydowii* is a response to hypersaline stress more than a response to salinity, as we have previously proposed [29].

### 3.4. Hog Phosphorylation Response to Osmotic Shock

Using several model organisms, it has been established that the HOG pathway coordinates responses to cellular osmotic stress. In *S. cerevisiae* and *D. hansenii,* the activation of this pathway leads to the transient phosphorylation of Hog upon a hyperosmotic shock [111]. In *H. werneckii,* the *hog* gene is duplicated while the protein phosphorylation dynamics is similar to Hog1p from *S. cerevisae* [18]. However, in *W. ichthyophaga,* the HOG system responds in the opposite direction, since the kinase is phosphorylated in the lowest salinities and is transiently dephosphorylated when the cells were exposed to a hypersaline medium [11,18,112]. These diverse signaling pathway configurations suggest that the HOG system is a key component of the mechanisms of adaptation to salinity and other stress-inducing conditions. Therefore, we studied the responses of the *A. sydowii* Hog orthologues to salinity stress.

**Figure 6:**
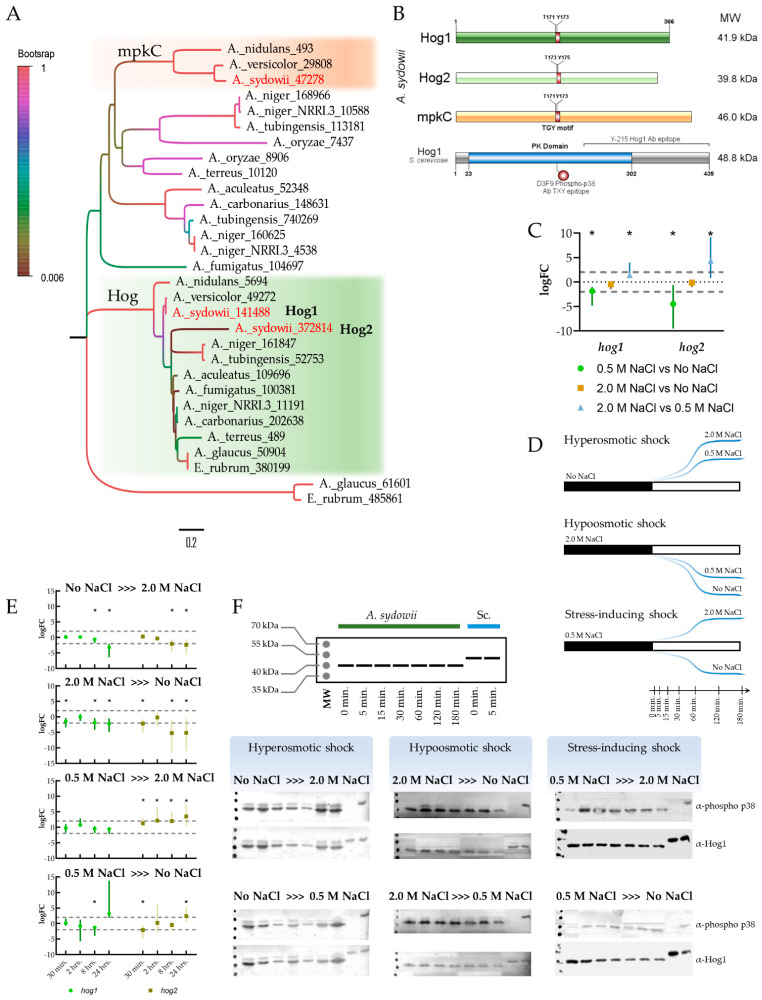
Hog MAPK system in *A. sydowii*. (**A**) Reconstruction of MAPK phylogeny in selected Aspergilli, including Hog1, Hog2, and MpkC genes of *A. sydowii.* A more extensive phylogenetic tree can be observed in Supplementary Figure S3. (**B**) Hog gene homologs on *A. sydowii* showing protein size, molecular weight, conserved phosphorylation motifs, protein kinase (PK) domain, and the region corresponding to Y-215 and D3F9 recognized by the antibodies used to detect phosphorylated and total Hog, respectively, by Western blot. (**C**) Relative expression of *Hog1* and *Hog2* transcripts in salt-adapted *A. sydowii* growing without NaCl, 0.5 M or 2.0 M NaCl, representing the average and standard deviations of three biological replicates (*n* = 3) and two technical qPCR replicates. (**D**) Diagram of the shock conditions used to test the phosphorylation dynamics of Hog1 and Hog2 (**E**) Relative expression of *Hog1* and *Hog2* transcripts after osmotic shock in *A. sydowii*, representing the average and standard deviations of three biological replicates (*n* = 3) and two technical qPCR replicates. The statistical significance (*) was assessed by a randomization test performed with the software Rest [46]. The dashed horizontal lines correspond to a cutoff logFC = ±2. (**F**) Phosphorylation of Hog MAPK homologs after different osmotic shocks. Extracts from *S. cerevisiae* cultures shifted from a medium without NaCl to a medium with 1.0 M NaCl were used as positive controls for Hog1 phosphorylation.

Few fungi, like several Aspergilli, have more than one copy of the Hog MAPK [113]. For example, *A. nidulans* has two orthologues of the *S. cerevisae* Hog1p, named SakA/HogA and MpkC. The former is responsible for most of the osmo-protective stress responses in *A. nidulans*, while the latter is involved in conidiation and oxidative stress response [113]. Interestingly, *A. sydowii* has a third copy of the Hog MAPK that cannot be found in its close relatives *A. versicolor* and *A. mulundensis*. Here we named these MAPKs as Hog1, Hog2, and MpkC, which respectively have protein identifiers 141488, 372814, and 47278 in the published *A. sydowii* CBS 593.65 genome (Figure 6A). All three orthologues have complete protein kinase (PK) domains and the conserved TGY phosphorylation site (Figure 6B), which implicates that they could be phosphorylated and biologically active. This allowed us to detect the phosphorylation of the *A. sydowii* MAPK orthologues using antibodies with cross-reactivity to the *S. cerevisiae* Hog1p.

The transcript levels of the *Hog1* variant might be higher than those of *Hog2*, according to previous RNA-seq experiments (Supplementary Figure S4) [29]. More important, the expression of both genes was similar under stress conditions (No NaCl or 2.0 M NaCl) but was lower under optimal salinity conditions (Figure 6C). This effect was more pronounced for *Hog2*, suggesting that this variant might play a more significant role in the salt stress response.

We have established that the conditions without NaCl or with 2.0 M NaCl induce stress responses in *A. sydowii* [29]. Therefore, changing the mycelium from media with 0.5 M NaCl to another condition induces stress responses, which might be different from hyperosmotic or hypoosmotic shock responses. A schematic representation of those experimental variations can be observed in Figure 6D. The differential expression of *Hog1* and *Hog2* under these scenarios showed that both genes were downregulated after hyper- or hypoosmotic shocks (Figure 6E), reducing the transcript abundance after long time periods (8 to 24 h post-stimulus). Again, this effect is more pronounced for *Hog2* gene variant than for *Hog1*. In contrast, *Hog2* gene expression is mostly upregulated after stress-inducing osmotic shocks, while *Hog1* expression is not significantly perturbed.

The phosphorylation of Hog MAPK variants was analyzed by Western Blot from 5 min to 3 h after an osmotic stimulus and compared to the phosphorylation dynamic of Hog1p in *S. cerevisiae* (Figure 6F). The independent patterns of Hog1/Hog2 phosphorylation could not be discerned with this experiment as the bands corresponding to both protein products overlapped. Conversely, a higher molecular weight band, below of *S. cerevisiae* Hog1p could correspond to the MpkC variant, according to its molecular weight prediction.

Both or at least one of the Hog1/Hog2 proteins were phosphorylated constitutively in the conditions without NaCl or in 2.0 M NaCl, and to a lesser extent in the 0.5 M NaCl optimal condition. Such phosphorylation status did not change upon hyper- or hypoosmotic shocks, but increased when cells were exposed to stress-inducing osmotic shocks considering the growth optimal condition. These changes were not transient, though, indicating that a sustained phosphorylation of Hog is the normal status of this signaling system in *A. sydowii*. Interestingly, MpkC protein was produced in detectable amounts only in the condition without NaCl where it was constitutively phosphorylated. It remained phosphorylated even after a hyperosmotic shock. In contrast, this protein was transiently phosphorylated upon hypoosmotic shock, especially from 2.0 M NaCl to the medium without NaCl.

The phosphorylation of Hog1p in *S. cerevisiae* and its homolog in *D. hansenii* occurs transiently and immediately after a mild stress stimulus, and persists up to half an hour in both models [54,114]. In contrast, in *D. hansenii* a prolonged Hog1 phosphorylation state was observed in cells subjected to a severe osmotic stress [54,114,115]. Interestingly, the phosphorylation of Hog1 remained after cells adapted to severe osmotic stress were subjected to a hypoosmotic shock [114,115].

Taken together, these results might indicate that the biological activity of Hog1/Hog2 is regulated transcriptionally, while regulation of their activity by phosphorylation is not evident, as they could not be discriminated by Western Blot. The HOG system in *A. sydowii* is responding to osmotic conditions that induce cellular stress, since the presence of salt in optimal concentrations did not seem to regulate transcript abundance or phosphorylation status of either of the Hog proteins. On the other hand, the Hog homolog MpkC was responsive to hypoosmotic shock. The interplay of these MAPKs on the regulation of salinity and stress responses should be studied further, as it does not follow the dynamics of other HOG systems in halotolerant or halophile model microorganisms.

### 3.5. Na^+^/K^+^ Ratio in A. sydowii

Eukaryotes also compensate osmotic imbalance through intracellular regulation of potassium and sodium ion levels [116], which has been as well observed in halophilic and halotolerant fungi [28,102,117,118,119]. Intracellular sodium ions are potentially toxic for eukaryotic cells, as they can inhibit numerous metabolic reactions and change cell membrane potential and transport systems [120,121]. Therefore, as the extracellular amount of sodium increases, the cells of halotolerant and halophile microorganisms increase the number of cation transporters to maintain a high K^+^/Na^+^ ratio [117,122]. Intracellular potassium is required in these conditions to sustain the potential across the plasma membrane, compensating for osmotic imbalance and the negative charges of macromolecules. Potassium is also involved in the regulation of protein synthesis and function [123,124].

**Figure 7:**
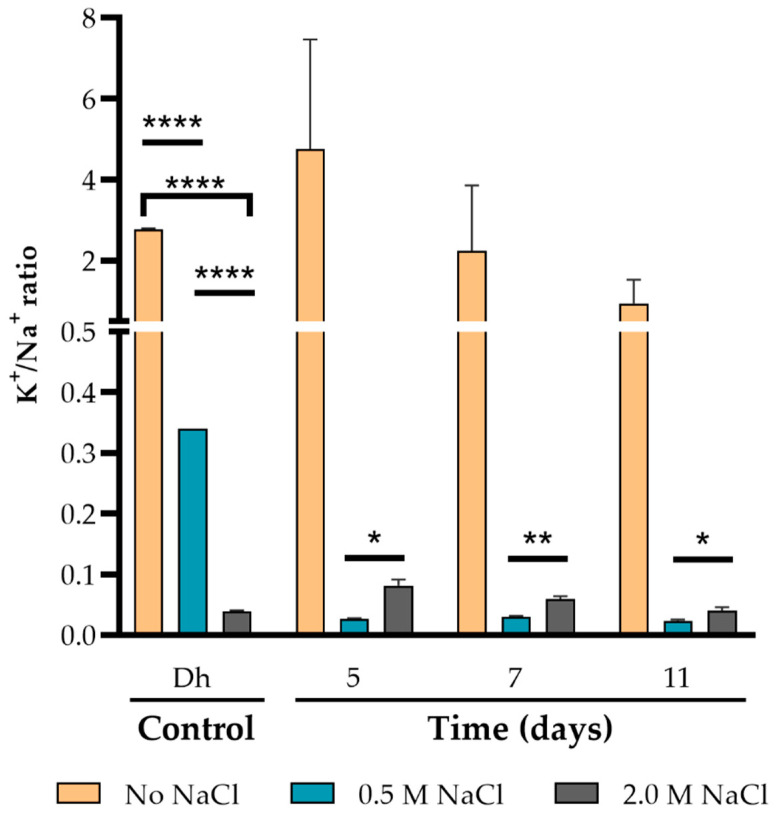
Regulation of intracellular K^+^/Na^+^ ratio in response to salinity in *A. sydowii* cells. Dh: *Debaryomyces hansenii* was used as a positive control. Data are means ± SD. Statistical significance was determined using the Holm-Sidak method, with alpha = 0.05. Each row was analyzed individually, without assuming a consistent SD. (* *p* < 0.05, ** *p* < 0.01, **** *p*< 0.0001).

In the halotolerant yeast *Debaryomyces hansenii*, used here as a control, the increase of extracellular sodium triggered the accumulation of potassium inside the cell. At 0.5 M NaCl, the K^+^/Na^+^ ratio was ten times smaller than in the media without NaCl (Figure 7). Increasing the extracellular concentration to 2.0 M NaCl, a four-fold increase with respect to the condition with 0.5 M NaCl, reduced the K^+^/Na^+^ ratio ten-fold. In contrast, *A. sydowii* does not seem to have the same compensatory mechanisms to regulate intracellular cation concentrations when growing in the medium with 0.5 M NaCl. In this condition the K^+^/Na^+^ ratio decreased almost twenty times compared to the medium without salt. A higher concentration of NaCl apparently triggered the accumulation of potassium or the removal of sodium, as the K^+^/Na^+^ ratio rose by two- to four-fold in the medium with 2.0 M NaCl compared to 0.5 M NaCl (Figure 7). This indicates that, as a consequence of salinity stress, the transport systems that regulate K^+^ uptake and Na^+^ extrusion were upregulated or activated [119], whereas this was not a requirement for the proper functioning of *A. sydowii* cells under optimal salinity. In this regard, this moderate halophile fungus has a different K^+^/Na^+^ accumulation pattern as compared to *S. cerevisiae* or other halotolerant fungi such as *D. hansenii* or *Debaryomyces nepalensis* when exposed to increasing concentrations of sodium [118].

### 3.6. Stress Induces Changes in the Redox State of A. sydowii

**Figure 8:**
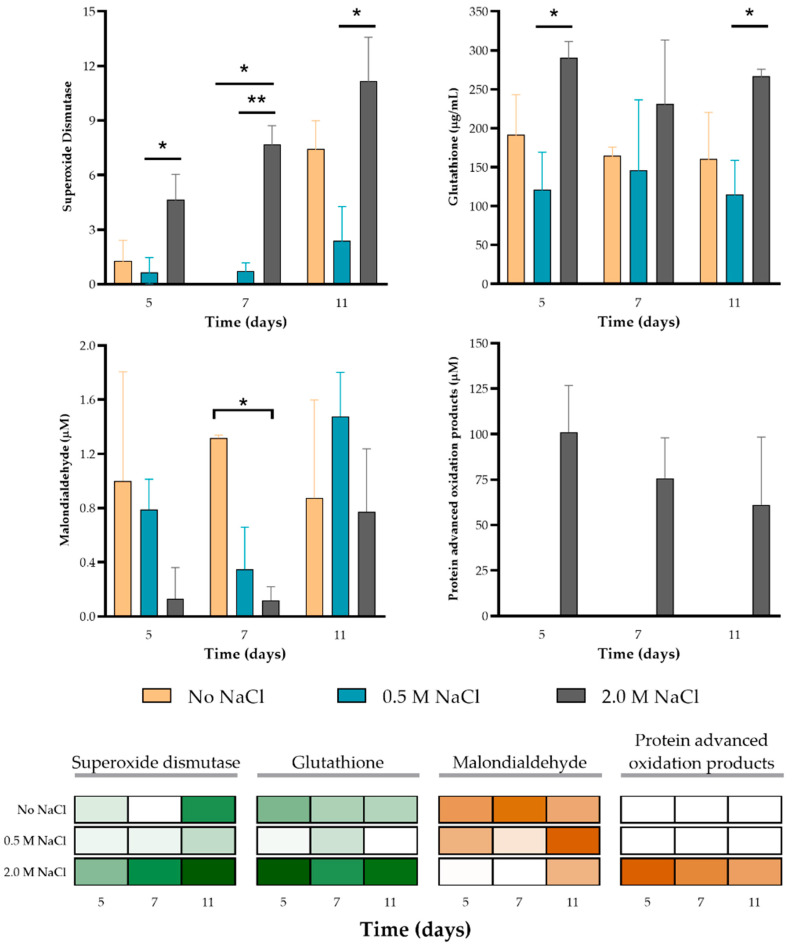
Oxidative stress and antioxidant markers evaluated in *A. sydowii* cultures under stable salinity conditions. Superoxide dismutase (SOD) and glutathione (GSH) (antioxidants markers; green in the heat maps), malondialdehyde (MDA) and protein advance oxidation products (PAOP) (oxidative damage markers; brown in the heat maps) were analyzed. Data are means ± SD. Bars with the asterisks (*) indicate the significant difference (** *p* < 0.01, * *p* < 0.05) between the control and its respective treated samples. Analyzed by a two-way ANOVA with Tukey’s multiple comparisons test.

Fungi and plants are prone to generate Reactive Oxygen Species (ROS) in the presence of a high salt concentration [125,126,127,128,129]. There is a causal link between salinity and potential oxidative stress, which is the result of the unbalanced generation vs. scavenging of ROS [128,129,130]. Whereas ROS are generated as normal by-products of aerobic metabolism, their accumulation alters the balance between oxidized and reduced glutathione. High ROS levels can damage macromolecules such as lipids, proteins, and nucleic acids, which can lead to cell death [131], although there is also evidence that ROS are signaling molecules that can regulate growth and cell differentiation [132,133]. However, to our knowledge, there is no evidence linking ROS levels and halophile fungi adaptation to extreme salinities. Therefore, we investigated oxidative and antioxidant responses of *A. sydowii* (lipoperoxidation, protein oxidation, GSH and SOD activity levels), under different salinity conditions. Hydroperoxides were not detected under our experimental conditions, perhaps because cells were already adapted at the tested times. As shown in Figure 8, SOD activity (percentage) and GSH concentration were higher when the fungus was growing in 2.0 M of NaCl reaching 11.2% on day 11 and 290.6 µg/mL on day 5, respectively (Figure 8). On day 11, SOD activity increased significantly (7.4%) when the fungus was growing without salt as compared with the optimal growing condition (0.5 M NaCl). Meanwhile, GSH was accumulated significantly at 2.0 M NaCl, with the lowest accumulation in the optimal growth condition (115–145 µg/mL) (Figure 8). The protein advanced oxidation products (PAOPs) were accumulated only in the hypersaline condition (2.0 M NaCl) reaching up to 101.2 µM on day 5, suggesting that this is the most stressful condition for *A. sydowii.* Malondialdehyde (MDA) accumulated at similar levels in all conditions by day 5 and 11. However, on day 7 there was an inverse correlation between MDA levels and the presence of salt. MDA is a byproduct of polyunsaturated fatty acid peroxidation mediated by free radicals or by lipoxygenases [134,135]. The heat map in Figure 8 shows that antioxidant mechanisms were reduced under optimal salinity as compared to hypoosmotic and hyperosmotic conditions. These results indicate that non-optimal salinity growth conditions (0 and 2.0 M NaCl) favor a higher production of ROS, in contrast to what is observed under optimal growth conditions (0.5 M NaCl). This is consistent with the detection of high levels of both antioxidants and oxidative markers in salt-tolerant bacterial isolates from a saline lake cultured in a media with 20% (3.4 M) NaCl and also with the resistance of *H. werneckii* to oxidative stress, which show a correlation between salinity and resistance to oxidative stress [136].

**Figure 9:**
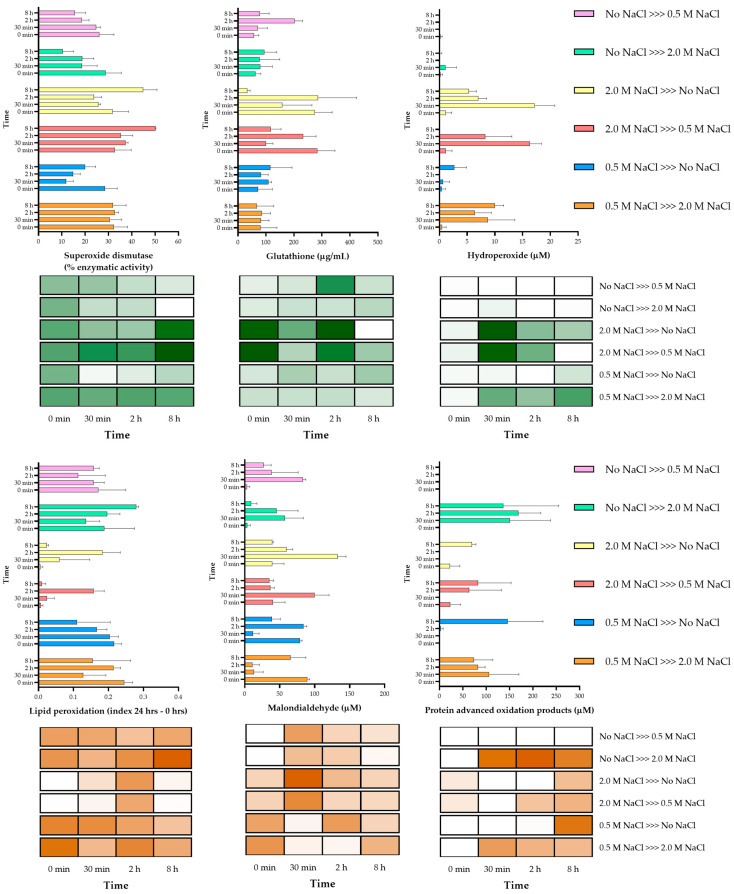
Oxidative stress and antioxidant markers evaluated in *A. sydowii* after osmotic shock. Superoxide dismutase (SOD) and glutathione (GSH) (antioxidants markers, green in the heat maps), malondialdehyde (MDA) and protein advance oxidation products (PAOP) (oxidative damage markers, red in the heat maps) were analyzed. Columns represents the average of at least three replicates, and bars represent the standard deviation. Details of statistical differences are shown in Supplementary Table S7.

Figure 9 shows *A. sydowii* dynamic response of oxidative markers and antioxidants when the fungus was challenged by osmotic stress. The fungus was grown for 7 days in MMG without salt, 0.5 M or 2.0 M of NaCl, then shifted to the other conditions and oxidative markers and antioxidants were evaluated after 30 min, 2, and 8 h (some time points were the same for the evaluation of compatible solutes and Hog phosphorylation). SOD activity and GSH content were similar when *A. sydowii* was growing without salt or with 0.5 M NaCl (time 0) and those levels were maintained when both conditions were shifted to 2.0 M NaCl. The levels of these antioxidant mechanisms were higher only when the fungus was growing in 2.0 M and they were maintained after shifting to media without salt or with 0.5 M NaCl. Hydroperoxides were not detected at the initial time in any condition, possibly because the fungus was already adapted to the growing media, while their levels increased mainly when *A. sydowii* was shifted to 2.0 M NaCl. These results correlate with PAOP levels, where noticeable changes were observed when the fungus was shifted from a media without salt or 0.5 M NaCl to media with 2.0 M NaCl. There was also an increase in PAOP levels in other conditions, but only after 8 h. After osmotic shock, hydroperoxide was detected at 30 min of the exposure to a new environment only when the starter culture was growing in 2.0 M NaCl. The hydroperoxide was also detected when the starter culture growing in 0.5 M salt was transferred to the hypersaline condition (2.0 M NaCl). Accordingly, protein oxidation was detected after 30 min of exposition to the hypo- or hypersaline media, while MDA levels had an inverse correlation with SOD activity and the GSH levels. When cells growing in the absence of salt were exposed to either 0.5 M or 2.0 M NaCl, they responded with a decrease in the enzymatic (SOD) and non-enzymatic (GSH) antioxidant mechanisms. Strikingly when the cells were exposed to the optimal Na^+^ condition, there was no accumulation of peroxide or oxidized protein. However, there was an increase in lipid peroxidation. In the conditions where the fungus was shifted to a higher salinity (2.0 M), there was more lipid peroxidation; however, this might be due to lipoxygenase (LOXs) activity, which is regulated by abiotic stresses, including high salinity [137], rather than to an unregulated accumulation of ROS. Under this condition, the fungal response was to maintain SOD activity with lower amounts of GSH (around 90 µg/mL).

Previously, whether measurements of antioxidant cellular systems are proper indicators of stress tolerance has been discussed [138]. The effect of oxidative stress can depend on the duplication rate of the fungus and other environmental conditions. The presence of antioxidant mechanisms is a positive indicator of cell’s tolerance to salinity [139]. ROS are not only indicators of stress, but also play regulatory roles depending on the cellular concentration. In plants, it has been proved that the expression of some LOXs is regulated by biotic and abiotic stresses including salinity and drought [137] and it is well known that ROS participate as signaling transduction molecules that control several pathways involved in the acclimation of these organisms to stressful conditions [140].

## 4. Conclusions

We have shown that *A. sydowii* displays unique physiological responses when grown under optimal and non-optimal salinity conditions. We found that 0.5 M NaCl, a salt concentration that is stressful for many other fungal species, results an optimal growing condition for *A. sydowii, a* salinity which did not trigger a stress response, and relied on the synthesis of compatible solutes to maintain the osmotic balance. Accordingly, we observed that glycerol-3-P dehydrogenase (*gpd*) gene induction and glycerol accumulation occur only as a response to saline stress, while other osmolytes are accumulated under optimal growth NaCl concentrations. The role of the constitutive phosphorylation of Hog kinase homologs in the accumulation of polyols observed under hypoosmotic conditions remains to be elucidated. However, it indicates a different regulation of the HOG pathway in moderate halophiles. Our results contribute to understand the response of the halophilic fungus *A. sydowii* under diverse saline environments. The establishment of 0.5 M NaCl as the optimal growth condition for this fungus is in accordance with its natural environment as a coral reef pathogen, as in the sea water NaCl concentration is around 0.6 M. This knowledge would also allow implementation of *A. sydowii* culture strategies for potential biotechnological applications, as this fungus is able to degrade hydrocarbons and lignocellulosic materials and can be a source of halotolerant enzymes.

## Supplementary Materials

The following are available online at https://www.mdpi.com/article/10.3390/jof7060414/s1, Figure S1: Chromatograms of all used standards. Concentration: 5 mg/mL each; Figure S2: Chromatograms of selected samples from all salinity conditions, Table S1: Primer information for qPCR analysis of enzyme genes involved in the synthesis of compatible solutes. Table S2: Growth kinetic parameters of *Aspergillus sydowii* grown at different NaCl concentrations. Table S3: *p* values from statistics analysis performed on experiments shown in Figure 1B. Figure S3: Reconstruction of MAPK phylogeny in selected Aspergilli, including Hog1, Hog2, and MpkC genes of *A. sydowii*. Table S4: Genes encoding compatible solute synthesis enzymes in *A. nidulans* and their homologue genes of in *A. sydowii.* Table S5: *p* values obtained from statistics analysis performed on experiments shown in Figure 4. Figure S4: Expression levels of *hog1* and *hog2* genes. Table S6: *p* values obtained in the statistics analysis performed on experiments shown in Figure 8. Table S7: *p* values obtained in the statistics analysis performed on experiments shown in Figure 9.
