# Supplementary material for: Osmolyte Signatures for the Protection of Aspergillus sydowii Cells under Halophilic Conditions and Osmotic Shock
Source: J Fungi (Basel). 2021 May 26;7(6):414. doi: 10.3390/jof7060414 (PMC8228332; doi:10.3390/jof7060414)
Supplement: Supplementary file 1 [file jof-07-00414-s001.zip › jof-1207251-supplementary.pdf]

Journal of *Fungi*

Supplementary Material

Osmolyte Signatures for the Protection of *Aspergillus sydowii* cells under Halophilic Conditions and Osmotic Shock

Eya Caridad Rodríguez-Pupo [1,2,†], Yordanis Pérez-Llano [1,2,†], José Raunel Tinoco-Valencia [3], Norma Silvia Sánchez [4], Francisco Padilla-Garfias [4], Martha Calahorra [4], Nilda del C. Sánchez [5], Ayixón Sánchez-Reyes [6], María del Rocío Rodríguez-Hernández [1], Antonio Peña [4], Olivia Sánchez [4], Jesús Aguirre [4], Ramón Alberto Batista-García [2], Jorge Luis Folch-Mallol [1] and María del Rayo Sánchez-Carbente [1,*]

[1] Centro de Investigación en Biotecnología, Universidad Autónoma del Estado de Morelos (UAEM), Av. Universidad 1001, Col. Chamilpa, Cuernavaca C.P. 62209, Morelos, México; eyarguez2013@gmail.com (E.C.R.-P.); yordanis.perezllano@yahoo.com (Y.P.-L.); rocio.rodriguez@uaem.mx (M.d.R.R.-H.); jordi@uaem.mx (J.L.F.-M.)

[2] Centro de Investigación en Dinámica Celular, IICBA, UAEM, Av. Universidad 1001, Col. Chamilpa, Cuernavaca, C.P. 62209, Morelos, México; rabg@uaem.mx

[3] Instituto de Biotecnología, Universidad Nacional Autónoma de México (UNAM), Campus Morelos, Av. Universidad 1001, Col. Chamilpa, Cuernavaca C.P. 62210, Morelos, México; raunel@ibt.unam.mx

[4] Instituto de Fisiología Celular, UNAM, Cto. Exterior s/n, Cd. Universitaria, Coyoacán, Cd. de México C.P. 04510, México; nsanchez@ifc.unam.mx (N.S.S.); fpadilla@ifc.unam.mx (F.P.-G.); mcalahor@ifc.unam.mx (M.C.); apd@ifc.unam.mx (A.P.) ; asanchez@ifc.unam.mx (O.S.); jaguirre@ifc.unam.mx (J.A.)

[5] Centro de Ciencias Genómicas, UNAM, Campus Morelos, Av. Universidad 1001, Col. Chamilpa, Cuernavaca CP62210, Morelos, México; nildita1985@gmail.com

[6] Catedras Conacyt-Instituto de Biotecnología, Universidad Nacional Autónoma de México (UNAM), Campus Morelos, Av. Universidad 1001, Col. Chamilpa, Cuernavaca C.P. 62210, Morelos, México; ayixon.sanchez@ibt.unam.mx

* Correspondence: maria.sanchez@uaem.mx

† These authors contributed equally to this work.

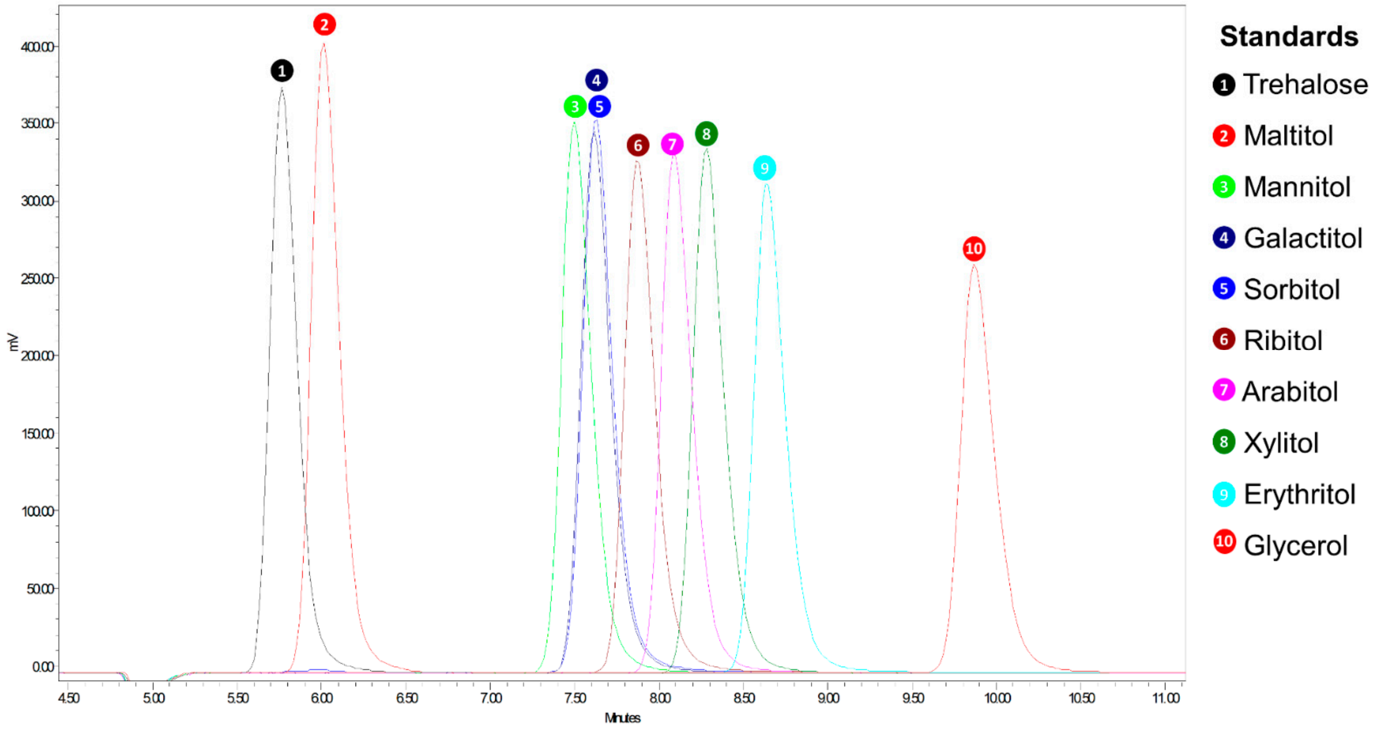

Figure S1. Chromatograms of all used standards. Concentration: 5 mg/mL each

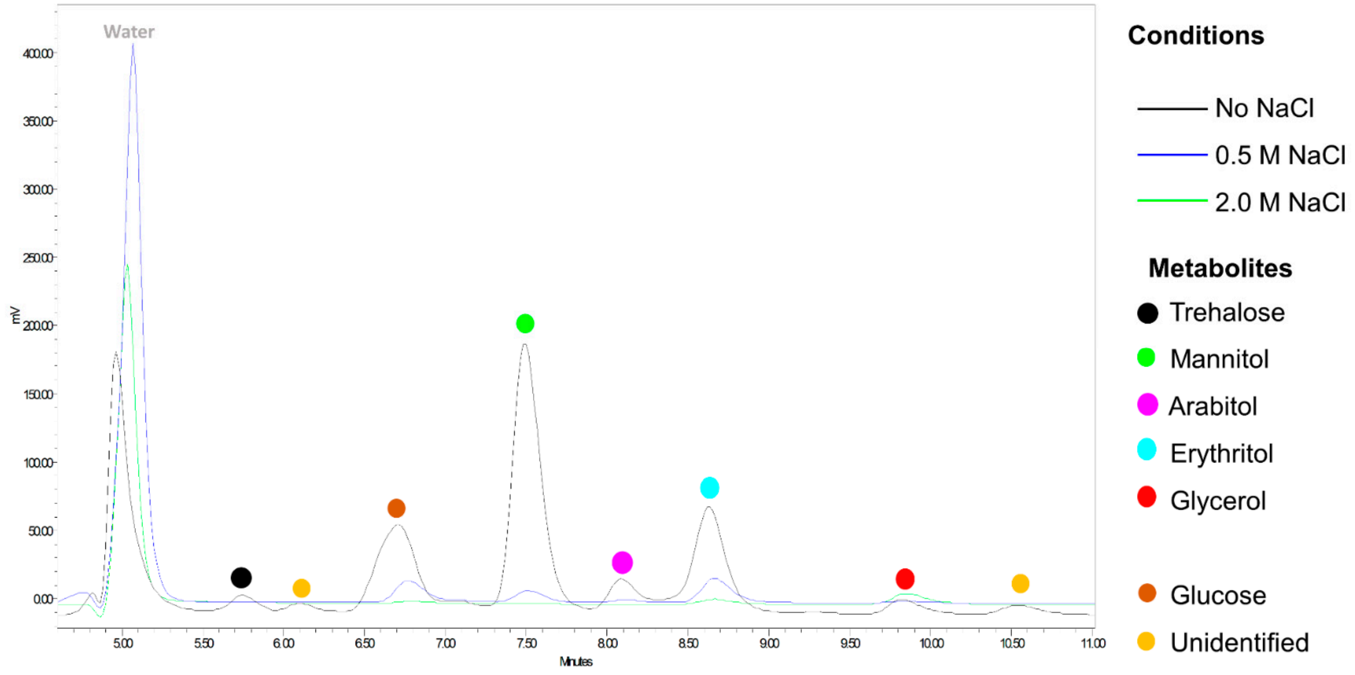

Figure S2. Chromatograms of selected samples from all salinity conditions. .

Table S1. Primer information for qPCR analysis of enzyme genes involved in the synthesis of compatible solutes.

Metabolite	Gene	Primer Sequence	Genome Location	Tm	Amp. size	Thib	Conc.	Eff.
Trehalose	*stps*	**Fw:** TCA ACG ATG GAC CAC TT	scaffold_10:397039-397058	60	109	63	300	0.92
		**Rv:** GGG TCG GAC TGC TTC GAT AT	scaffold_10:396950-396969					
	ccg-9	**Fw:** GTC AGC CAC CCT GTG AAA AC	scaffold_3:1753499-1753518	60	84	65	300	1.03
		**Rv:** CAG CCA ATC TGT CGA TGC AG	scaffold_3:1753435-1753454					
Mannitol	*mtld*	**Fw:** CAT CAA CGC CAC CGA TAC AC	scaffold_5:597303-597322	60	97	63	300	0.92
		**Rv:** ACA TTG AGG CCG TTG TTT GG	scaffold_5:597185-597204					
	m2dh	**Fw:** GCA TTG TCT CGC TGA CCA TC	scaffold_3:2943327-2943346	60	128	63	300	1.00
		**Rv:** GGT GGT ACG GGG AGT CTT TT	scaffold_3:2943435-2943454					
Erythritol	*tktB*	**Fw:** GCC ACC CAT CGC AGT ATC TA	scaffold_14:515665-515684	60	126	63	700	0.96
		**Rv:** TGA TAG TCT CGA CCA TGG CG	scaffold_14:515559-515578					
	tktA	**Fw:** TGC CTT CCA GCA GAT TCA GT	scaffold_2:2845201-2845220	60	126	65	300	1.09

		**Rv:** CGT ATG GCC GTT TTC CCA TC	scaffold_2:2845095-2845114					
	tad	**Fw:** CTC ATC TCT CCC TTC GTC GG	scaffold_2:1207666-1207685	60	100	65	300	0.95
		**Rv:** TCT GGA CAG ACT TGA CAC CG	scaffold_2:1207746-1207765					
Glycerol	*gpd*	**Fw:** ACA GGC TCT ACC CTC TGA GT	scaffold_2:1570111-1570130	60	124	60	500	1.06
		**Rv:** GAG AGC GCC TCC CAA TGA TA	scaffold_2:1570215-1570234					
HOG	*hog1*	**Fw:** GTC TTT GCC CTT CCC ATT TTC	scaffold_1:1249970-1249990	60	150	60	500	0.92
		**Rv:** GAA TTC CGC CAT TTT GAC CG	scaffold_1:1249841-1249860					
	hog2	**Fw:** CAT GAA TAC CTC GCT CCG TAC	NA	60		63	500	0.96
		**Rv:** TCA TCT TCC AAG TAT GCA CCG	scaffold_1:4867322-4867342					

Table S2. Growth kinetic parameters of *Aspergillus sydowii* grown at different NaCl concentrations.

NaCl Concentration (M)	Specific Growth Rate (μ)(h^{-1})	Doubling Time (t_d)(h)
No NaCl	0.0514	13.48
0.5	0.0683	10.14
1.0	0.0629	11.01
2.0	0.0474	14.62

Table S3. p values from statistics analysis performed on experiments shown in Figure 1B. Statistical differences were evaluated by one way ANOVA with Kruskal-Wallis and Dunn´s multiple comparisons test where $p < 0.05$ is statistically significant.

Solutes	Condition	*p* Value		
		Day 5	Day 7	Day 11
Trehalose	No NaCl vs. 0.5 M NaCl	0.5172	0.0504	0.019
	No NaCl vs. 2.0 M NaCl	0.5172	0.0504	0.5172
	0.5 M NaCl vs. 2.0 M NaCl	0.019	>0.9999	0.5172
Mannitol	No NaCl vs. 0.5 M NaCl	0.5391	0.5172	0.5172
	No NaCl vs. 2.0 M NaCl	0.5391	0.019	0.019
	0.5 M NaCl vs. 2.0 M NaCl	0.0219	0.5172	0.5172
Arabitol	No NaCl vs. 0.5 M NaCl	0.5391	0.5172	0.5391
	No NaCl vs. 2.0 M NaCl	0.5391	0.5172	0.5391
	0.5 M NaCl vs. 2.0 M NaCl	0.0219	0.019	0.0219
Erythritol	No NaCl vs. 0.5 M NaCl	0.0219	0.5172	0.5172
	No NaCl vs. 2.0 M NaCl	0.5391	0.5172	0.5172
	0.5 M NaCl vs. 2.0 M NaCl	0.5391	0.019	0.019
Glycerol	No NaCl vs. 0.5 M NaCl	>0.9999	>0.9999	>0.9999
	No NaCl vs. 2.0 M NaCl	0.3062	0.0504	0.0504
	0.5 M NaCl vs. 2.0 M NaCl	0.0306	0.0504	0.0504

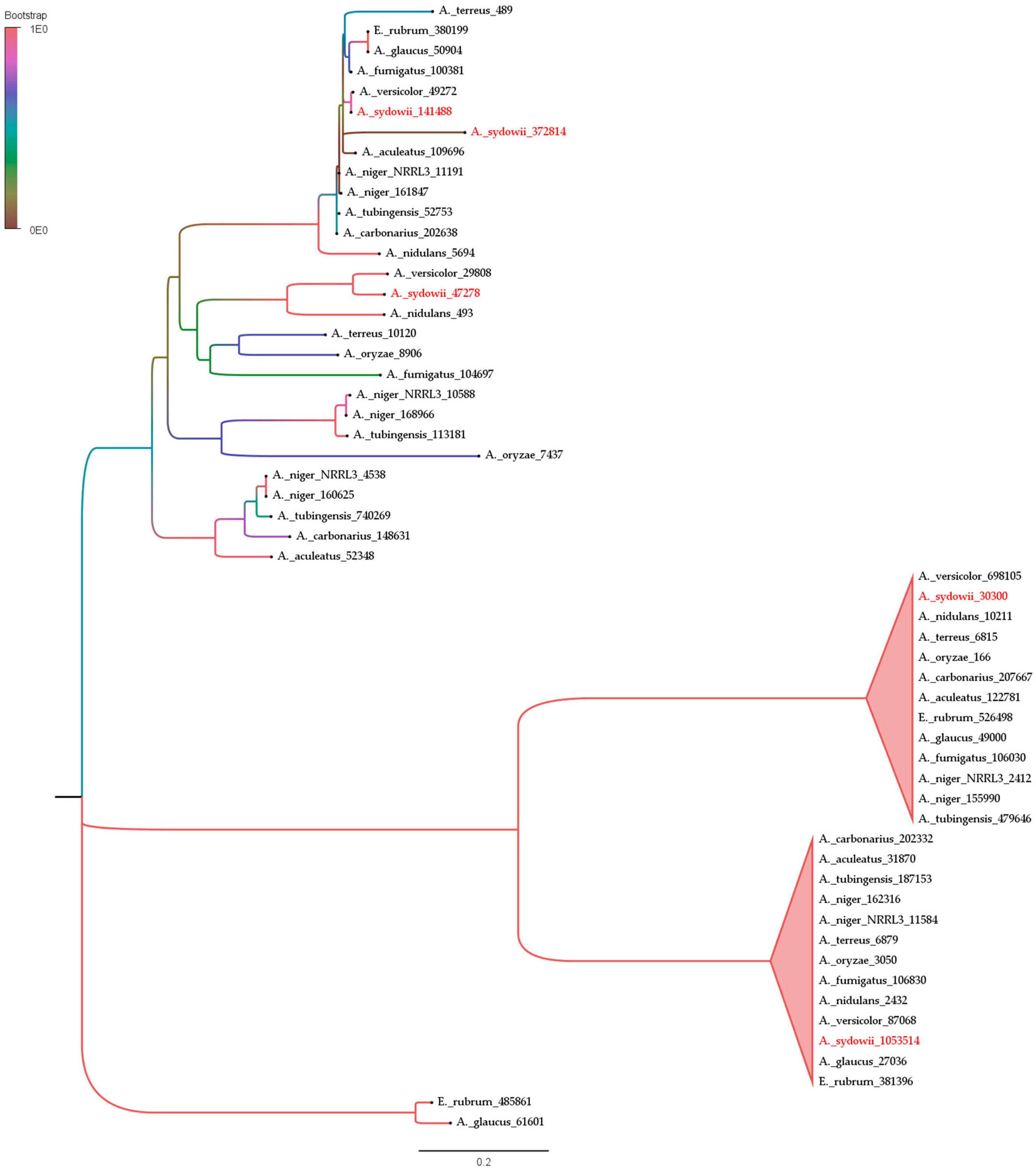

Figure S3. Reconstruction of MAPK phylogeny in selected *Aspergilli*, including Hog1, Hog2, and MpkC genes of *A. sydowii*.

Table S4. Genes encoding compatible solute synthesis enzymes in *A. nidulans* and their homologue genes of in *A. sydowii.*

Gene	Enzyme	*A. sydowii*		
		**Gene ID ***	**Protein ID ***	**% Ident ****
		**Trehalose Pathway**		
tpsA	Trehalose phosphate synthase subunit	e_gw1.4.743.1	149182	95
orlA	Trehalose phosphate phosphatase subunit	fgenesh1_pm.3_#_972	56871	85
tps3	TPS regulatory subunit	fgenesh1_pm.7_#_385	59235	89
stps	Heat shock trehalose phosphate synthase	fgenesh1_pm.10_#_111	60104	50
ccg-9	Trehalose phosphate synthase	CE565934_14846	780972	83
tpp	Trehalose-6-P phosphatase	gm1.12758_g	96075	87
treb	Neutral tre-halase	e_gw1.4.354.1	149568	90
trea	Acid tre-halase	fgenesh1_pm.18_#_129	62558	84
		**Mannitol Pathway**		
mtld	Mannitol-1-phosphate 5-dehydrogenase	e_gw1.5.2043.1	152275	86
m2dh	Mannitol 2-dehydrogenase	fgenesh1_pg.3_#_1082	29920	84
mpp	Manitol/Hexitol phosphatase			
hk	Hexokinase			
		**Glycerol Pathway**		
gpd	Glycerol-3-P dehydrogenase	fgenesh1_kg.2_#_690_#_Locus2578v1rpkm69.02	40910	89
gpd-m	Glycerol-3-P dehydrogenase mitochondrial	fgenesh1_pm.1_#_8	52965	79
gpp	Glycerol-3-P phosphatase	fgenesh1_kg.1_#_238_#_Locus2655v1rpkm66.24	38002	91
gut1	Glycerol-kinase	e_gw1.4.2272.1	149605	92
		gm1.5577_g	88894	83
had1	Halo-acid dehalogenase			
dak1	Dihydroxyacetone kinase			
gld1	Glycerol dehydrogenase	CE68380_3389	283418	90
		fgenesh1_pg.5_#_204	31279	86
		fgenesh1_pm.11_#_239	60569	56
		**Pentose phosphate pathway (Arabitol and Erythritol)**		
tktA	Transketolase	e_gw1.2.510.1	142846	95
tktB	Transketolase - putative	gm1.10640_g	93957	90
tad	Transaldolase	fgenesh1_pm.2_#_386	55064	94
larA / xylA	L-arabinose/D-xylose reductase	e_gw1.2.1363.1	144547	87
ardh	L-arabinitol dehydrogenase	CE897698_12019	1112736	80

* As in the sequenced genome in Mycocosm ** % Identity to *A. nidulans* homologue gene.

Table S5. *p* values obtained from statistics analysis performed on experiments shown in Figure 4. ($p < 0.05$ is statistically significant, one-way ANOVA and Dunnett´s multiple comparison test).

Solutes	Condition	*p Value*					
		No NaCl-0.5M NaCl	No NaCl-2.0M NaCl	2.0M NaCl-No NaCl	2.0M NaCl-0.5M NaCl	0.5 M NaCl-No NaCl	0.5 M NaCl-2.0M NaCl
Trehalose	0 min vs. 10 min	0.9484	<0.0001	<0.0001	0.6028	0.9998	<0.0001
	0 min vs. 30 min	0.9929	<0.0001	<0.0001	0.3974	0.993	<0.0001
	0 min vs. 2 h	0.417	<0.0001	<0.0001	0.2554	0.7194	<0.0001
	0 min vs. 8 h	0.6234	<0.0001	<0.0001	0.1114	0.1675	<0.0001
	0 min vs. 24 h	0.397	<0.0001	<0.0001	0.237	<0.0001	<0.0001
	0 min vs. 48 h	0.8485	<0.0001	<0.0001	<0.0001	0.9925	<0.0001
Mannitol	0 min vs. 10 min	0.0045	<0.0001	0.0004	>0.9999	0.9997	<0.0001
	0 min vs. 30 min	<0.0001	<0.0001	<0.0001	>0.9999	0.9996	<0.0001
	0 min vs. 2 h	<0.0001	<0.0001	0.0021	>0.9999	<0.0001	<0.0001
	0 min vs. 8 h	<0.0001	<0.0001	<0.0001	<0.0001	<0.0001	<0.0001
	0 min vs. 24 h	<0.0001	<0.0001	<0.0001	<0.0001	0.0004	<0.0001
	0 min vs. 48 h	<0.0001	<0.0001	<0.0001	>0.9999	0.0002	<0.0001
Arabitol	0 min vs. 10 min	0.4791	<0.0001	<0.0001	>0.9999	0.7318	<0.0001
	0 min vs. 30 min	0.0148	<0.0001	0.47	>0.9999	0.9997	<0.0001
	0 min vs. 2 h	<0.0001	<0.0001	>0.9999	>0.9999	<0.0001	<0.0001
	0 min vs. 8 h	0.0052	<0.0001	0.0065	<0.0001	0.0327	<0.0001
	0 min vs. 24 h	0.8105	<0.0001	<0.0001	<0.0001	0.0857	<0.0001
	0 min vs. 48 h	<0.0001	<0.0001	<0.0001	>0.9999	0.2647	<0.0001
Erythritol	0 min vs. 10 min	0.1093	<0.0001	<0.0001	0.0001	0.7	<0.0001
	0 min vs. 30 min	0.0471	0.0354	<0.0001	<0.0001	0.9997	<0.0001
	0 min vs. 2 h	<0.0001	0.9979	<0.0001	<0.0001	0.9997	<0.0001
	0 min vs. 8 h	0.0039	<0.0001	<0.0001	0.3936	<0.0001	<0.0001
	0 min vs. 24 h	<0.0001	0.0149	0.0022	<0.0001	0.0001	<0.0001
	0 min vs. 48 h	<0.0001	<0.0001	<0.0001	<0.0001	<0.0001	<0.0001
Glycerol	0 min vs. 10 min		>0.9999	<0.0001	<0.0001	>0.9999	>0.9999
	0 min vs. 30 min		>0.9999	<0.0001	<0.0001	>0.9999	>0.9999
	0 min vs. 2 h		>0.9999	<0.0001	<0.0001	>0.9999	<0.0001
	0 min vs. 8 h		<0.0001	<0.0001	<0.0001	<0.0001	<0.0001
	0 min vs. 24 h		<0.0001	<0.0001	<0.0001	0.0005	<0.0001
	0 min vs. 48 h		<0.0001	<0.0001	<0.0001	0.0011	<0.0001

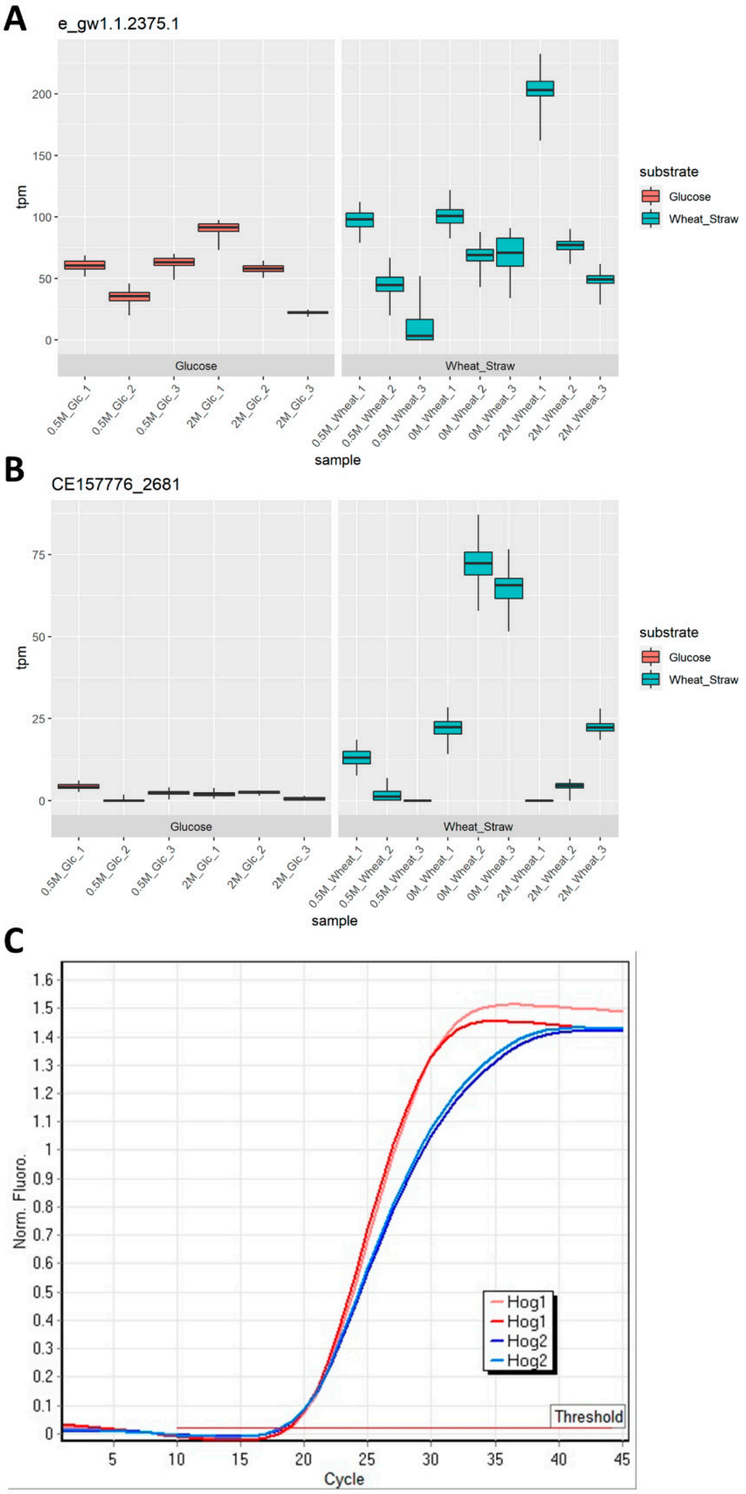

Figure S4. Expression levels of *hog1* and *hog2* genes. Expression of *hog1* **(A)** and *hog2* **(B)** genes measured by RNA-Scheme 1. Amplification curves examples of hog1 and hog2 (**C**); (red, Cq = 18.8) and Hog2 (blue, Cq = 18.3) measured by qPCR in a pooled cDNA sample from all tested conditions (0 M, 0.5 M, and 2.0 M NaCl);.

Table S6. *p* values obtained in the statistics analysis performed on experiments shown in Figure 8.

Oxidative Markers	Condition	*p* Value		
		Day 5	Day 7	Day 11
	No NaCl vs. 0.5 M NaCl	0.7352	0.1955	0.0518
SOD	No NaCl vs. 2.0 M NaCl	0.0708	0.0108	0.1890
	0.5 M NaCl vs. 2.0 M NaCl	0.0425	0.0050	0.0186
	No NaCl vs. 0.5 M NaCl	0.3008	0.9327	0.5803
GSH	No NaCl vs. 2.0 M NaCl	0.1212	0.4840	0.1567
	0.5 M NaCl vs. 2.0 M NaCl	0.0288	0.5106	0.0429
	No NaCl vs. 0.5 M NaCl	0.9049	0.0587	0.4844
MDA	No NaCl vs. 2.0 M NaCl	0.3419	0.0029	0.9782
	0.5 M NaCl vs. 2.0 M NaCl	0.0504	0.5400	0.2067
	No NaCl vs. 0.5 M NaCl	N/A	N/A	N/A
PAOP	No NaCl vs. 2.0 M NaCl	N/A	N/A	N/A
	0.5 M NaCl vs. 2.0 M NaCl	N/A	N/A	N/A

Table S7. Statistics analysis performed on experiments shown in Figure 9.

	p Value					
**Condition**	**SOD**	**GSH**	**HPO**	**Lipid Peroxidation**	**MDA**	**PAOP**
No NaCl >>> 0.5 M NaCl						
0 min. vs. 30 min.	0.9638	0.9807	0.9989	0.9763	<0.0001	>0.999
0 min. vs. 2 h	0.1319	*0.0061	0.9989	0.3731	*0.0184	>0.9999
0 min. vs. 8 h	*0.0225	0.9261	0.9989	0.9763	0.1502	>0.9999
No NaCl >>> 2.0 M NaCl						
0 min. vs. 30 min.	*0.0317	0.9649	0.8895	0.4522	*0.0002	*0.0003
0 min. vs. 2 h	*0.034	0.9739	0.9986	0.9958	*0.0042	<0.000
0 min. vs. 8 h	<0.0001	0.8172	>0.9999	0.0898	0.9445	*0.0012
2.0 M NaCl >>> No NaCl						
0 min. vs. 30 min.	0.2782	*0.035	<0.0001	0.4448	<0.0001	0.8622
0 min. vs. 2 h	0.114	0.9903	*0.0016	*0.0003	0.2527	0.8622
0 min. vs. 8 h	*0.0038	<0.0001	*0.032	0.9593	>0.9999	0.4226
2.0 M NaCl >>> 0.5 M NaCl						
0 min. vs. 30 min.	0.499	*0.0005	<0.0001	0.9558	<0.0001	0.8519
0 min. vs. 2 h	0.8543	0.5544	*0.0002	*0.0021	0.9834	0.5478
0 min. vs. 8 h	*0.0001	*0.0016	0.8085	0.9996	0.9626	0.2454
0.5 M NaCl >>> No NaCl						
0 min. vs. 30 min.	*0.0002	0.7412	0.998	0.9782	<0.0001	>0.9999
0 min. vs. 2 h	*0.0026	0.9923	0.9859	0.4957	0.9605	0.9996
0 min. vs. 8 h	0.0835	0.6404	0.3535	*0.0373	*0.006	*0.0005
0.5 M NaCl >>> 2.0 M NaCl						
0 min. vs. 30 min.	0.9434	>0.9999	<0.0001	*0.0201	<0.0001	0.0128
0 min. vs. 2 h	0.9993	0.9996	*0.0015	0.8123	<0.0001	0.0672
0 min. vs. 8 h	0.9991	0.9797	<0.0001	0.0898	0.1462	0.1118

* $p < 0.05$ is statistically significant using a two-way ANOVA and Dunnett´s multiple comparison test.
